# Identification of autophagy-associated circRNAs in sepsis-induced cardiomyopathy of mice

**DOI:** 10.1038/s41598-023-38998-7

**Published:** 2023-07-21

**Authors:** Ming-zhi Zheng, Jun-sheng Lou, Yun-peng Fan, Chun-yan Fu, Xing-jia Mao, Xiang Li, Kai Zhong, Lin-huizi Lu, Lin-lin Wang, Ying-ying Chen, Liang-rong Zheng

**Affiliations:** 1 Department of Cardiology and Atrial Fibrillation Center, The First Affiliated Hospital, School of Medicine, Zhejiang University, Hangzhou, China; 2 Department of Pharmacology, Hangzhou Medical College, Hangzhou, 310053 Zhejiang China; 3 Department of Orthopedic Surgery, The First Affiliated Hospital, Zhejiang University School of Medicine, Hangzhou, 310003 China; 4 Department of Basic Medicine Sciences, and Department of Obstetrics of the Second Affiliated Hospital, Zhejiang University School of Medicine, Hangzhou, 310009 Zhejiang China; 5 Department of Basic Medicine Sciences, and Department of Orthopaedics of Sir Run Run Shaw Hospital, Zhejiang University School of Medicine, Hangzhou, 310016 China; 6 Department of Clinical Medicine, Hangzhou Medical College, Hangzhou, 310053 Zhejiang China

**Keywords:** Biotechnology, Cardiology, Diseases, Medical research

## Abstract

Circular RNAs (circRNAs) play a role in sepsis-related autophagy. However, the role of circRNAs in autophagy after sepsis-induced cardiomyopathy (SICM) is unknown, so we explored the circRNA expression profiles associated with autophagy in an acute sepsis mouse model. At a dose of 10 mg/kg, mice were intraperitoneally administered with lipopolysaccharides. The myocardial tissue was harvested after 6 h for microarray analysis, qRT-PCR, and western blotting. Gene Ontology, Kyoto Encyclopedia of Genes and Genomes and Gene Set Enrichment Analysis were evaluated, and a competing endogenous RNA network was constructed, to evaluate the role of circRNAs related to autophagy in SICM. In total, 1,735 differently expressed circRNAs were identified in the LPS-treated group, including 990 upregulated and 745 downregulated circRNAs. The expression level of the autophagy-specific protein p62 decreased, while the ratio of LC3 II to LC3 I increased. Additionally, 309 mRNAs and 187 circRNAs were correlated with autophagy in myocardial tissue after SICM. Of these, 179 circRNAs were predicted to function as “miRNA sponges”. Some distinctive circRNAs and mRNAs found by ceRNA analysis might be involved in autophagy in SICM. These findings provide insights into circRNAs and identified new research targets that may be used to further explore the pathogenesis of SICM.

## Introduction

Sepsis has a high annual incidence and often causes acute dysfunction of multiple organs. In particular, sepsis-induced cardiac dysfunction maintains a high mortality^1,2^. The pathogenesis of sepsis is complex and involves the inflammatory cascade response, oxidative stress, mitochondrial dysfunction, calcium overload, autophagy, and apoptosis^3–9^.

Circular RNA (circRNA) molecules are a general feature in gene expression programs in human cells and were first identified in 2012^10^. Tey et al. revealed that some circRNAs act as molecular sponges that bind and seal microRNAs^11^. The role of circRNAs has been studied in the pathogenesis of various diseases, e.g., cancer, spinal cord injury, and vascular diseases^12–16^. The role of circRNAs has also been reported in multiple organ damage triggered by sepsis. For example, the circRNA HIPK3 aggravates sepsis-induced acute kidney injury by modulating the microRNA-338^17^. However, there are few studies on the function of circRNAs in sepsis-induced cardiomyopathy (SICM).

Autophagy is a process that involves phagocytosis of the cytoplasmic protein or organelle of the cell itself, which are coated into the vesicles and fuse with the lysosome to form autolysosomes that degrade their wrapped contents. Recent studies have shown that circEXOC5-related signal cascade regulates inflammation and autophagy, and aggravates sepsis-induced acute lung injury^18^. It is poorly understood whether autophagy is modulated by circRNAs in SICM.

In this study, we analyzed circRNA expression profiles in SICM in a mice model to predict the autophagy-related circRNAs and mRNAs in SICM based on the circRNA-miRNA-mRNA network.

## Materials and methods

### Animals and study design

We conducted all animal experiments following the Guide for the Care and Use of Laboratory Animals published by the US National Institutes of Health (8th edition, NRC 2011). This study was approved by the Experimental Animal Ethics Committee of Zhejiang University (ZJU201305-1-02-047). Forty male C57BL/6 mice (7–8 weeks and 20–25 g, equivalent to adults at the ages of about 20 years old) were provided by the Zhejiang University Laboratory Animal Research Center. Mice had free access to food and water in cages at 23 °C and a 12-h light/dark cycle.

Mice were randomly divided into two groups and received intraperitoneal injections of lipopolysaccharides (LPS, Darmstadt) or normal saline at a dose of 10 mg/kg as described by our previous study^19^. According to the inclusion and exclusion criteria, the mice who died within 6 h after injection should be excluded from the study. In the present study, no mice died within 6 h after injection, so all mice treated with LPS or saline were used for further analysis in the study. After 6 h of injection, mice in each group were intraperitoneally injected with 1% pentobarbital sodium (40 mg/kg) for anesthesia and sacrificed by cervical dislocation. In total, eight mice hearts from two groups were immediately removed and placed in liquid nitrogen. The expressions of circRNAs and mRNAs in the samples were evaluated by Shanghai Biotechnology Corporation. Four additional myocardial samples per group were tested using western blotting. Sixteen mice hearts from two groups were collected to measure superoxide dismutase (SOD) activity and malondialdehyde (MDA) content. Another four left ventricle samples per group were collected to evaluate myocardial ultrastructure.

### Assessment of the myocardial injury

According to the commercial assay kits (Haimen), SOD activity and MDA content in the heart samples which had been homogenized and lysed in lysis buffer were measured.

Left ventricular samples were fixed in 2.5% glutaraldehyde solution and 1% osmic acid. After being dyed with 4% uranyl acetate solution, the myocardial tissues were dehydrated with alcohol and acetone. Then, the samples were embedded and sliced, stained with 5% Uranium acetate staining and lead citrate. Changes of myocardial ultrastructure were observed under a transmission electron microscopy.

### RNA extraction and purification

According to the method mentioned in our previous work, RNA was extracted, identified, and purified from the left ventricle of all mice heart samples^19^. The reagents used included Takara RNAiso Plus (Mountain View), NucleoSpin RNA Clean-up XS kit (Düren), and RNase-Free DNase Set (QIAGEN).

### Microarray analysis

Similar to our earlier work, we used Cy-3 to label the amplified total RNA^19^. The labeled cRNAs were purified and hybridized. Agilent Microarray Scanner was used to scan the array slides of circRNAs and mRNAs. We used Quantile algorithm and limma packages in R software to normalize the raw data. The reagents used included Low Input Quick Amp Labeling Kit, Gene Expression Hybridization Kit and Wash Buffer Kit (Santa Clara), and RNeasy mini kit (QIAGEN).

### Quantitative real-time polymerase chain reaction

Similar to the previously reported method, qRT-PCR was performed to confirm circRNA expression^19^. The reagents used included ReverTra Ace qPCR kit (Tokyo) and Capacity cDNA Reverse Transcription Kit (ABI).

### Bioinformatics analysis

Normalized signal values were calculated using the log2 method. The circRNAs (fold change > 2, *p* < 0.05) after SICM damage were evaluated. To explore the potential function of circRNAs, especially of the autophagy-related circRNAs, we performed relevant functional and bioinformatics analyses using the method reported by Yao Ying^20^. We used websites for correlation analyses, including Gene Ontology (http://www.geneontology.org), Kyoto Encyclopedia of Genes and Genomes (https://www.genome.jp/kegg/), miRDB (http://www.mirdb.org/), and Cytoscape (https://cytoscape.org/)^21–23^.

### Western blot (WB) analysis

The left ventricles from mice myocardial tissue (LPS or control group, n = 4 per group) were homogenized in RIPA lysis solution (Beyotime) to prepare the sample for western blotting. We examined the contents of the autophagy landmark proteins p62 and microtubule-associated protein 1 light chain 3 (LC3) using the method reported by Lou Junsheng^24^. The reagents used included cocktail (Sigma-Aldrich), BCA Protein Assay Kit (Thermo Fisher Scientific), p62 (Abcam), LC3 (Cell Signaling Technology), β-actin, and secondary antibodies (Santa Cruz Biotechnology).

### Gene set enrichment analysis (GSEA)

GSEA was performed to identify the markedly enriched gene set clusters in myocardial tissue. The enrichment score (ES) curve was built using the GSEA4.3.1 software. The gene set with False Discovery Rate (FDR) < 0.25, |normalized enrichment scores (NES)|> 1, and nominal (NOM) *p* value < 0.05 was recognized as statistically significant.

### Statistical analysis

All data were shown as mean ± standard deviation. GraphPad Prism 9.0 was used for analysis. Normality test was performed by using Shapiro–Wilk test. Student’s *t* test was used to compare significance between two groups. *P* < 0.05 was considered statistically significant.

## Results

### CircRNA expression profiles in myocardium of septic mice

After LPS injection, mouse hearts displayed an increased MDA content and a decreased SOD activity (Fig S1). Meanwhile, the mitochondrion bloated and bubbled, and cristae was disrupted in the mice myocardial treated with LPS (Fig. S1). All the above results indicated that the animal model of sepsis-induced cardiomyopathy was established.

**Figure 1:**
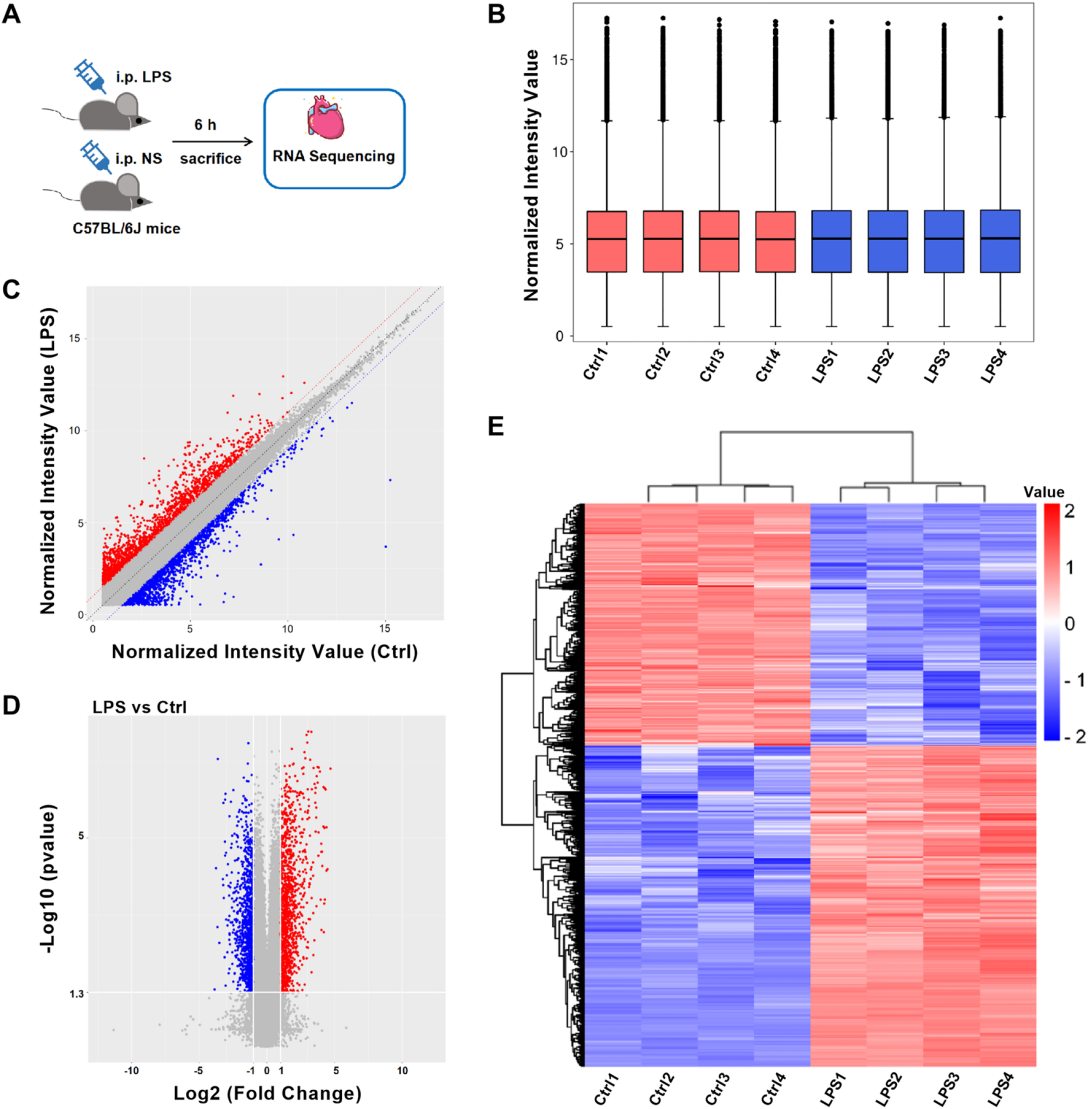
Expression profiles of circRNAs in the mouse myocardium after LPS injection. (**A**) Experimental design for RNA sequencing. (**B**) The box plot shows the distribution of circRNA expression profiles. (**C**–**E**) The scatter plot, volcano plot, and heatmap show the differentially expressed circRNAs. Red and blue colors represent upregulated and downregulated circRNAs, respectively. LPS, lipopolysaccharides; Ctrl, control; circRNA, circular RNA.

**Figure 2:**
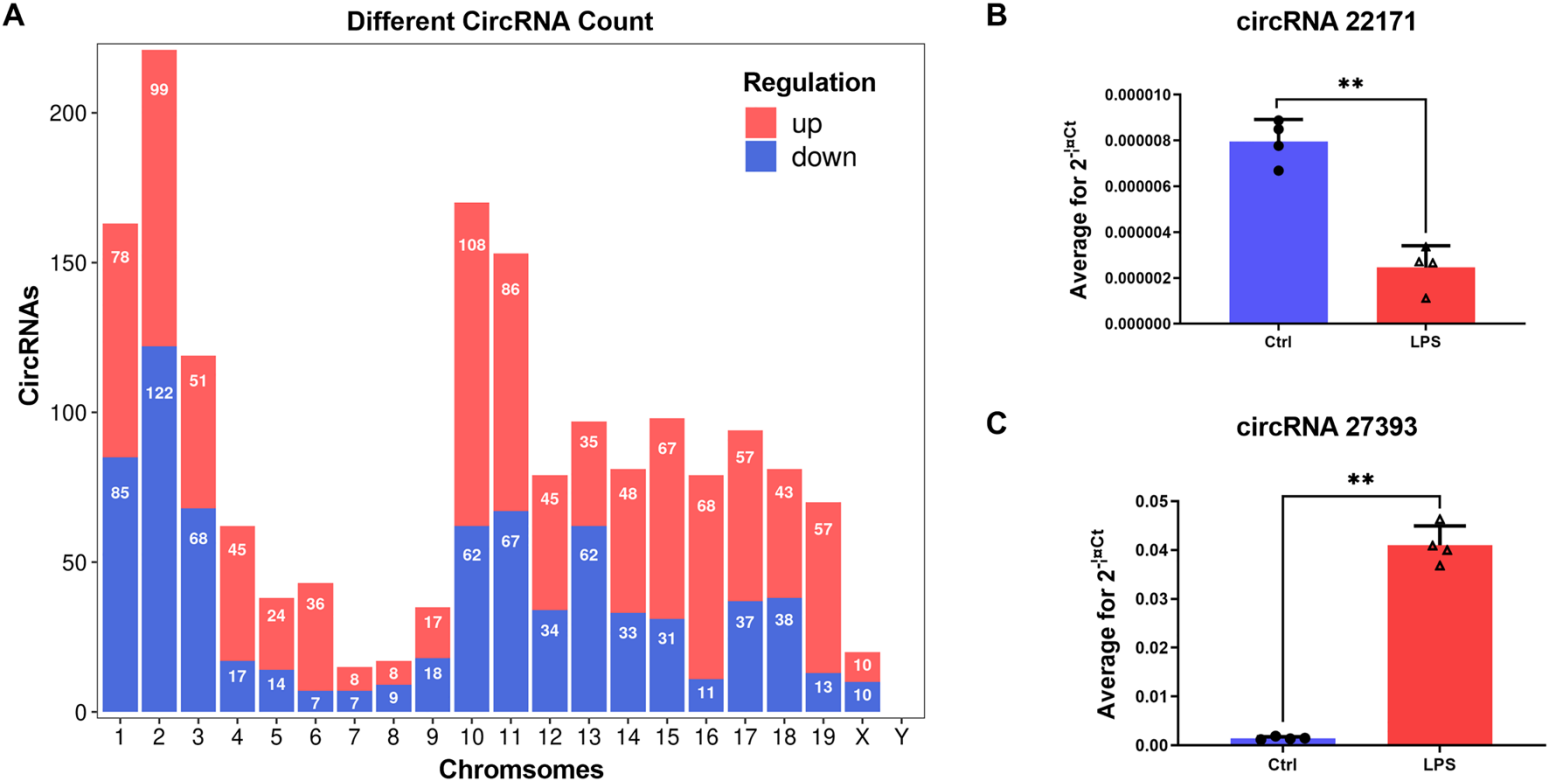
Distribution of altered circRNAs and their validation. (**A**) The distributions of dysregulated circRNAs in mouse chromosomes. (**B**, **C**) qRT-PCR verification of two circRNAs (circRNA.22171 and circRNA.27393). Data are presented as mean ± SD (n = 4). **p < 0.01 *vs.* control group.

We performed RNA sequencing on mouse hearts (Fig. 1A). The distribution of circRNA expression profiles in all samples showed good symmetry and dispersion (Fig. 1B). Differentially expressed circRNAs (DE circRNAs) were illustrated in red or blue color (Fig. 1C, D). Red color represents twofold upregulation of circRNAs, while blue color represents twofold downregulation of circRNAs in Fig. 1C. In Fig. 1D, circRNAs with fold change ≥ 2 (*p* < 0.05) and those with fold change ≤ 0.05 (*p* < 0.05) are shown in red or blue color, respectively. The expression features of dysregulated circRNAs were evaluated (Fig. 1E). In the LPS group vis-à-vis the control group, 1,735 circRNAs were differentially expressed (fold change > 2, *p* < 0.05), including 990 upregulated (57.06%) and 745 downregulated (42.94%) circRNAs. Depending on the degree of the fold change, the top 20 DE circRNAs are listed in Table 1. The genomic locations of the 1735 dysregulated circRNAs transcribed from all chromosomes, except chromosomes X and Y, are shown in Fig. 2A.

**Table 1:** The top 20 differentially expressed CircRNAs in the myocardium after LPS injection.

circRNA	Fold change	Regulation	Chromosome	Strand	Host Gene	*p* value
cicRNA.19315	25.98361958	Up	chr9	+	Casp4	2.18 × 10^–7^
cicRNA.2986	22.32013373	Up	chr17	−	Fkbp5	5.58 × 10^–5^
cicRNA.27393	21.23872246	Up	chr2	−	Pfkfb3	5.52 × 10^–6^
mmu_circ_0006655	21.06661119	Up	chr17	−	Fkbp5	6.04 × 10^–5^
cicRNA.2985	20.18851181	Up	chr17	−	Fkbp5	6.84 × 10^–7^
cicRNA.2983	19.42937517	Up	chr17	−	Fkbp5	2.98 × 10^–4^
cicRNA.321	19.37133487	Up	chr19	+	Cd274	6.43 × 10^–7^
cicRNA.3756	19.04363807	Up	chr16	−	Nfkbiz	5.67 × 10^–6^
cicRNA.2982	18.84151186	Up	chr17	−	Fkbp5	3.72 × 10^–4^
cicRNA.2984	18.46545391	Up	chr17	−	Fkbp5	1.81 × 10^–3^
cicRNA.15200	13.54572201	Down	chr10	+	Ptprb	5.28 × 10^–5^
cicRNA.15202	12.57297813	Down	chr10	+	Ptprb	1.36 × 10^–3^
mmu_circ_0015638	12.38524172	Down	chr9	+	Fam55d	1.27 × 10^–7^
cicRNA.27243	11.45238239	Down	chr2	+	Stard9	3.26 × 10^–3^
cicRNA.14922	9.564336752	Down	chr10	+	Arhgap18	9.67 × 10^–5^
cicRNA.25961	9.384905328	Down	chr2	+	Etl4	1.34 × 10^–4^
cicRNA.22171	9.279374752	Down	chr4	−	Car8	1.44 × 10^–6^
cicRNA.8088	9.110753037	Down	chr13	−	Rasgrf2	2.73 × 10^–4^
cicRNA.27242	8.752751778	Down	chr2	+	Stard9	7.22 × 10^–3^
cicRNA.8799	8.591014207	Down	chr13	+	Cmah	1.22 × 10^–5^

### Altered CircRNA expression was confirmed by real-time PCR

Among the top 20 DE circRNAs, two circRNAs were randomly selected for qRT-PCR. The primers of circRNAs and GAPDH are shown in Table 2. Consistent with the microarray results, circRNA.27393 was significantly upregulated, while circRNA.22171 was significantly downregulated (*vs.* control group, *p* < 0.05; Fig. 2B–C).

**Table 2:** Primers used for qRT-PCR of circRNAs.

Gene	Primer name	Sequence (5’–3’)
GAPDH (Mouse)	Gapdh-F	TCCTGCACCACCAACTGCTTAG
	Gapdh-R	AGTGGCAGTGATGGCATGGACT
circRNA_27393	CUST_98850_PI435794180-F	TGTCACCAGGCTGTTCTACGC
	CUST_98850_PI435794180-R	CAAGTCCCTGCACTCTTGTCG
circRNA_22171	CUST_91041_PI435794180-F	CAGCGAAGGAGTTACCTGGATATT
	CUST_91041_PI435794180-R	CCTCCTGACAAGACTGCATCTG

### DE CircRNA function analysis

**Figure 3:**
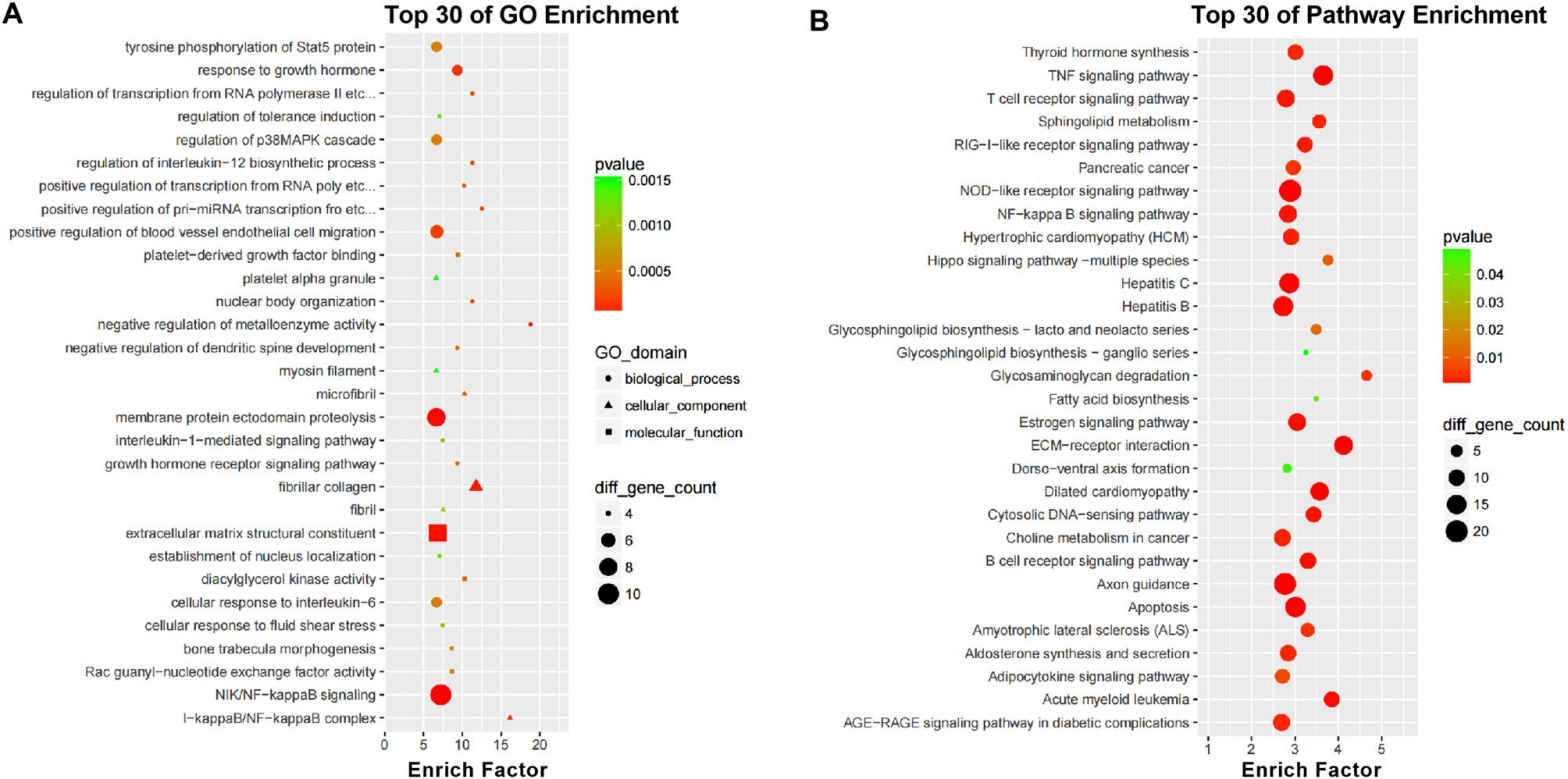
GO and KEGG enrichment analyses of differentially expressed circRNAs. (**A**) Top 30 enriched GO terms. (**B**) Top 30 enriched KEGG pathways.

The potential functions of 1735 DE circRNAs were predicted by GO and KEGG enrichment analyses, and the results are shown in bubble charts (Fig. 3A–B). Based on the size of the enriched factors, the top 30 most remarkably enriched GO items were selected (Fig. 3A). The results showed that the host genes of DE circRNAs during LPS treatment were mostly involved in the “negative regulation of metalloenzyme activity” and “I-kappa B/NF-kappa B complex” (Fig. 3A). The KEGG pathway for enrichment analysis indicated that most of the host-genes of DE circRNAs were related to glycosaminoglycan degradation and ECM-receptor interaction pathways (Fig. 3B).

### CeRNA network prediction and annotation

**Figure 4:**
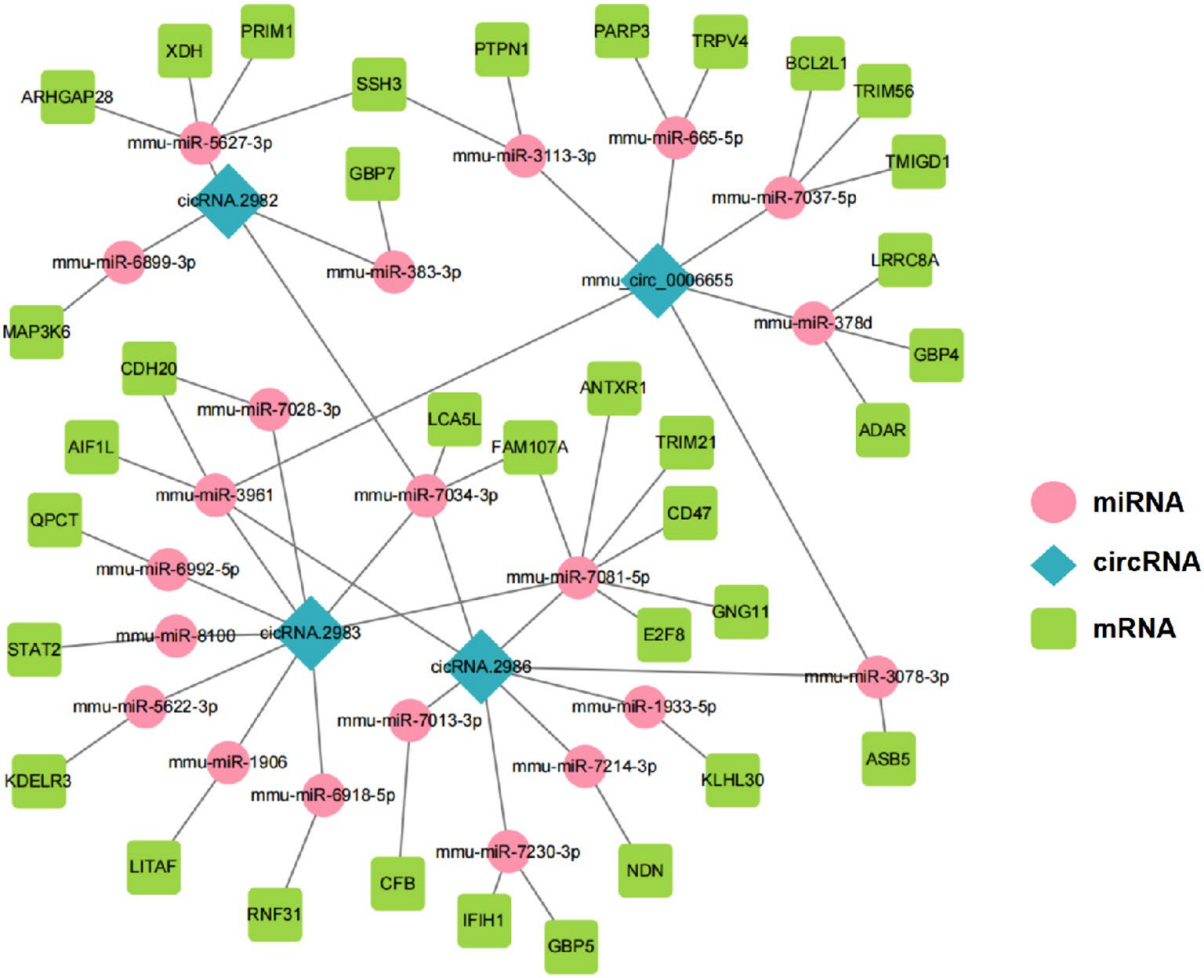
CeRNA analysis of mouse myocardium after LPS injection. CircRNAs, miRNAs, and mRNAs are showed as blue diamonds, pink ellipses, and green rectangles, respectively. ceRNA, competing-endogenous RNA; miRNA, microRNA; mRNA, messenger RNA.

The functions of circRNA include competitive adsorption of microRNAs (miRNA), regulation of RNA-binding proteins, and modulation of variable cleavage or transcription. CircRNAs bind to the corresponding miRNAs by MREs, which act as “sponges” preventing miRNA binding to the target gene and then jointly participating in the expression regulation of the target genes. This mechanism of action is called the “ceRNA mechanism”. This is the main research concept related to circRNAs. We filtered the DE circRNAs shown in Table 1 and found four associated mRNAs using ceRNA analysis: circRNA.2982, circRNA.2983, circRNA.2986, and mmu_circ_0006655. The result is shown in Fig. 4 and shows that the ceRNA mechanism exists after SICM.

### Autophagy-related CircRNA prediction

**Figure 5:**
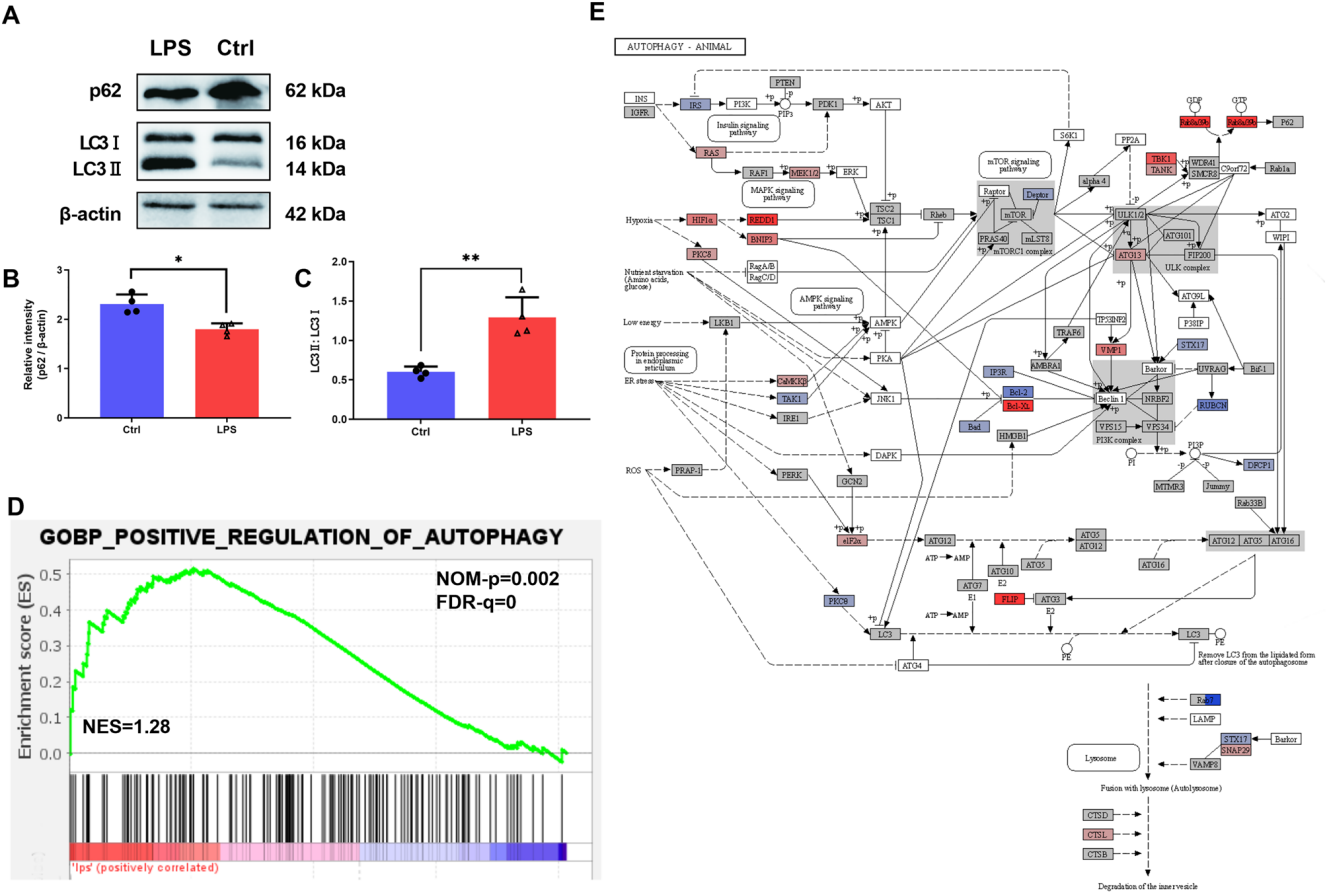
Detection of autophagy in mouse myocardium after LPS injection. (**A**–**C**) Western blotting analysis for p62 protein expression and the ratio of LC3 II to LC3 I. *p < 0.05, **p < 0.01 *vs.* control group. Data are presented as mean ± SD. n = 4. (**D**) Positively enriched gene sets identified by GSEA. NES, NOM p value, and FDR are shown. (**E**) KEGG map04140 shows the autophagy-animal pathway (https://www.kegg.jp/kegg/kegg1.html)^21–23^. Red and blue colors represent gene upregulation and downregulation, respectively. GSEA, Gene Set Enrichment Analysis; NES, Normalized Enrichment Scores; NOM, Nominal; FDR, False Discovery Rate.

**Figure 6:**
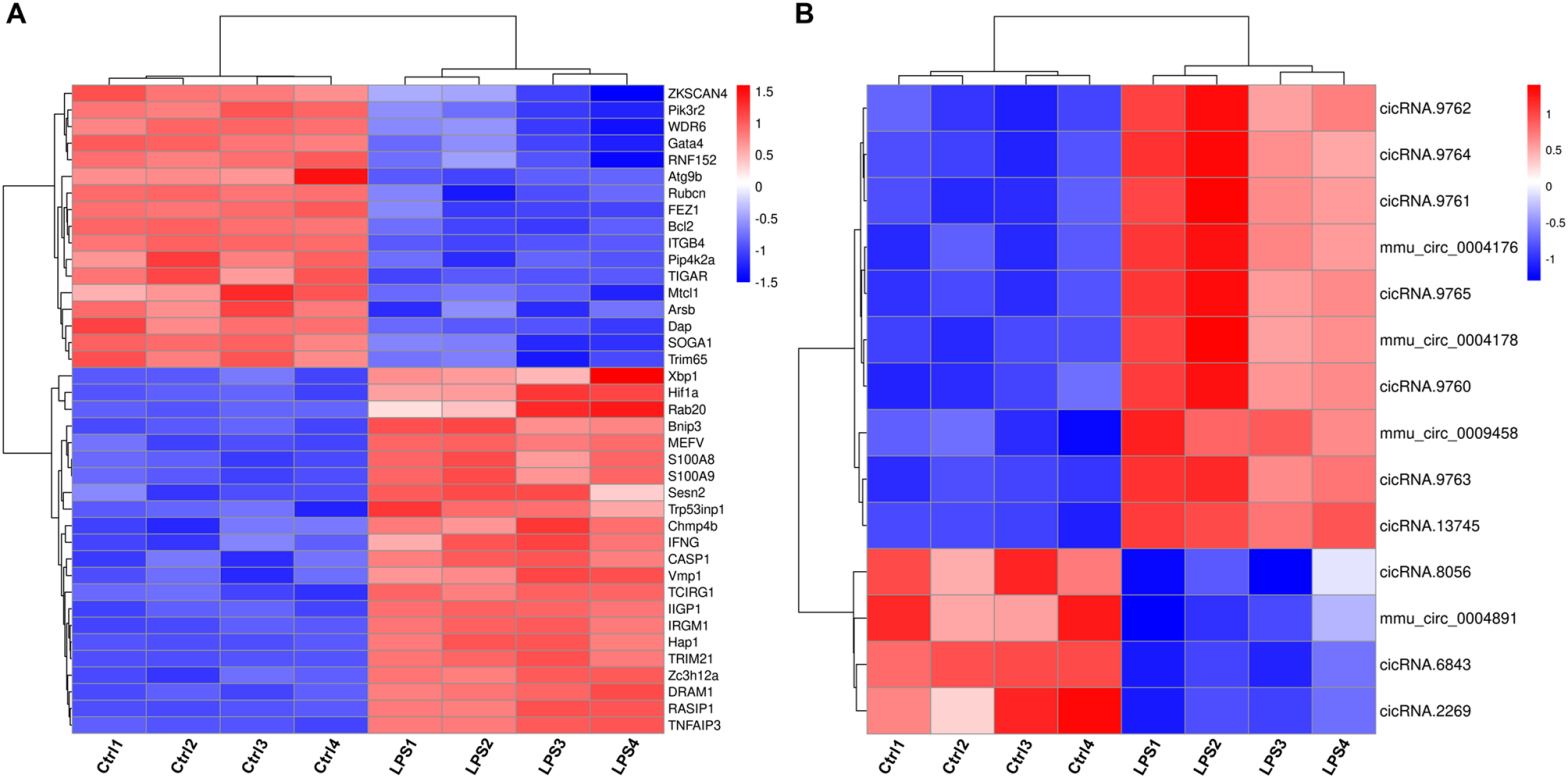
GO analysis of mRNAs and circRNAs related to autophagy in mouse myocardium after LPS injection. (**A**) Heat map showing differentially expressed mRNAs based on the GO analysis of autophagy. (**B**) Heat map showing differentially expressed circRNAs based on the GO analysis of autophagy-related mRNAs.

**Figure 7:**
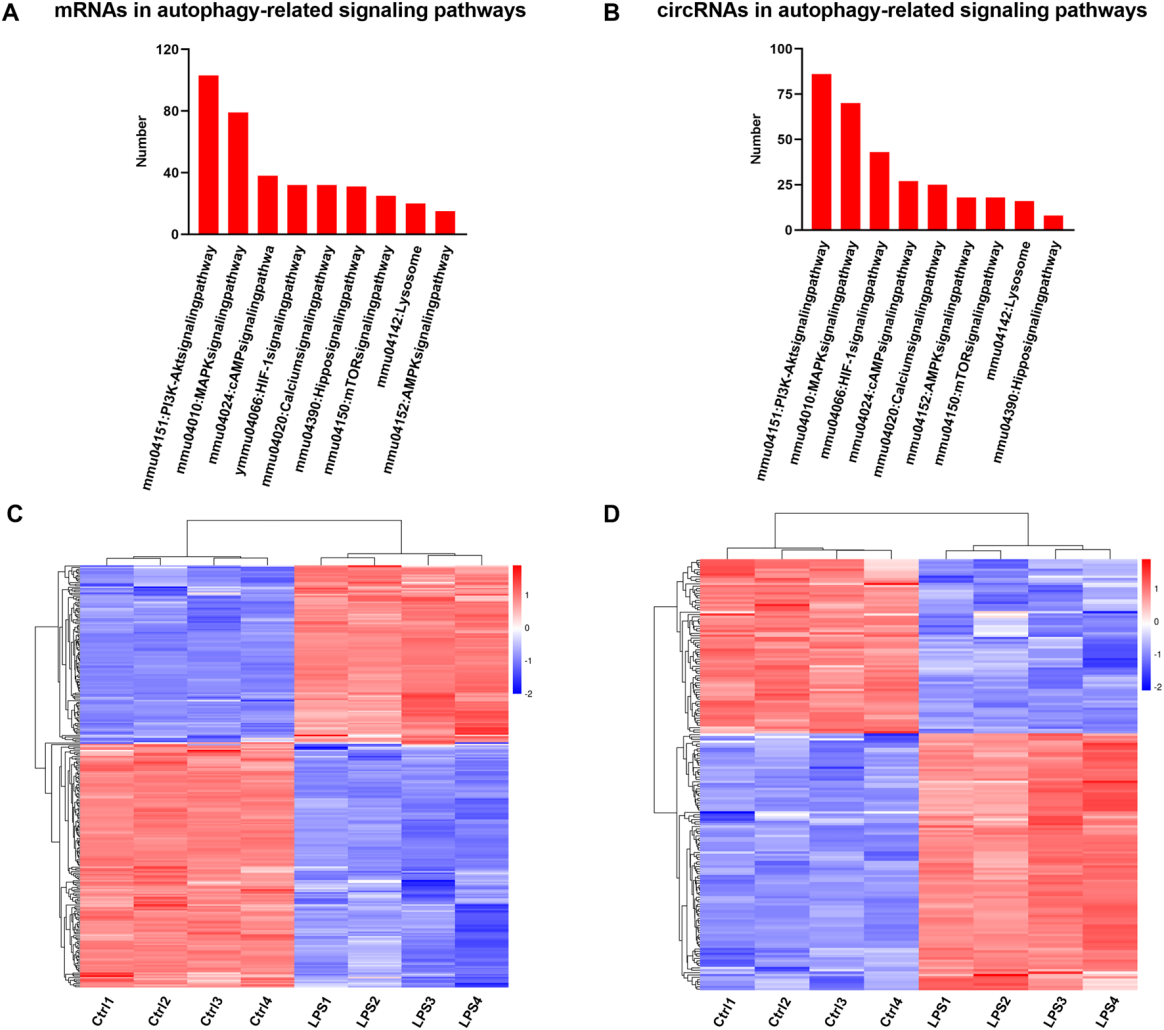
KEGG analysis of mRNAs and circRNAs related to autophagy in mouse myocardium after LPS injection. (**A**, **B**) The number of differentially expressed mRNAs and circRNAs in the prediction of autophagy-related KEGG signaling pathways. (**C**) Heat map showing differentially expressed mRNAs based on the KEGG analysis of autophagy. (**D**) Heat map showing differentially expressed circRNAs based on the KEGG analysis of autophagy-related mRNAs.

We explored the SICM-induced autophagy-related circRNAs. First, to confirm the protein expression of autophagy markers in mouse myocardium after SICM, we performed western blotting to detect the levels of proteins p62 and LC3 (Fig. 5A). Compared to the control group, the LPS group saw a decrease in the expression of p62 protein (1.80 ± 0.12, *p* < 0.05, Fig. 5B) as well as a significant increase in the ratio of LC3 II to LC3 I (1.60 ± 0.38, *p* < 0.01, Fig. 5C), suggesting the occurrence of autophagy. GSEA indicated that the gene set related to positive regulation of autophagy was enriched in SICM damage (Fig. 5D). The results further confirmed that autophagy occurs in mouse cardiomyocytes after 6 h of LPS treatment. In the animal autophagy signaling pathway (KEGG: mmu04140), several circRNA-related host genes were altered after SICM, with some of them (e.g., REDD1, FLIP, Bcl-XL, and TBK1) upregulated and others (e.g., RAB7B and RUBCN) downregulated (Fig. 5E). Next, we predicted the pathways associated with autophagy via KEGG pathway analysis and found nine autophagy-associated endogenous signaling pathways (Fig. 7A–B). The mRNAs were predominantly enriched in the PI3K-Akt (n = 103) and MAKP (n = 79) signaling pathways (Fig. 7A). The circRNAs were also predominantly enriched in the PI3K-Akt (n = 86) and MAKP (n = 70) signaling pathways (Fig. 7B). Third, based on the nine autophagy-related signaling pathways, GO analysis revealed that 39 mRNAs (fold change > 2, *p* < 0.05) were correlated with biological processes of autophagy, such as “autophagy (GO: 0006914)”, “regulation of autophagy (GO: 0010506)”, “negative regulation of autophagy (GO: 0010507)”, and “positive regulation of autophagy (GO: 0010508)” (Fig. 6A). Additionally, 14 circRNAs related to these mRNAs were found and were forecasted to be associated with autophagy (Fig. 6B). KEGG analysis revealed that 279 autophagy-correlated mRNAs (fold change > 2, *p* < 0.05) were enriched in the same nine autophagy-related signaling pathways (Fig. 7C, Table S1). Furthermore, ceRNA network prediction showed that 183 circRNAs were correlated with these mRNAs (Fig. 7D, Table S2). Finally, among these circRNAs, 179 autophagy-related circRNAs have binding sites on miRNAs based on the prediction of MREs (Table S3). Then, we found the corresponding target genes using Forecast websites. The top 10 upregulated autophagy-related circRNAs, based on the fold-change size, were circRNA.27393, circRNA.27392, circRNA.5564, circRNA.5562, mmu_circ_0005739, circRNA.5566, circRNA.5563, circRNA.27348, circRNA.5573, and circRNA.27394. Based on the top 10 circRNAs, we applied the miRDB websites and explored the mRNAs associated with autophagy. In Table 3, we listed the autophagy-related mRNAs that bind to the corresponding miRNAs with the highest target score. Most mRNAs were predominantly involved in the Hippo, PI3K-Akt, and mTOR signaling pathways.

**Table 3:** The top 10 differently expressed circRNAs involved in LPS-induced autophagy.

circRNA	Fold change	Regulation	miRNA	Target gene	Downstream pathways or biological proecsses
circRNA.27393	21.24	Up	mmu-miR-1933-3p	Dap	Autophagy
			mmu-miR-448-5p	Rnf152	Autophagy mTOR signaling pathway
			mmu-miR-125a-5p	Ajuba	Hippo signaling pathway
			mmu-miR-125b-5p	Ajuba	Hippo signaling pathway
circRNA.27392	12.80	Up	mmu-miR-1903	Vav3	cAMP signaling pathway
			mmu-miR-669f.-5p	Bcl2	Autophagy PI3K-Akt signaling pathway HIF-1 signaling pathway
			mmu-miR-770-3p	Thbs1	PI3K-Akt signaling pathway
			mmu-miR-671-5p	Arrb1	MAPK signaling pathway
			mmu-miR-705	Laptm5	Lysosome
circRNA.5564	10.77	Up	mmu-miR-105	Hspa1b	MAPK signaling pathway
			mmu-miR-5114	Gli2	Hippo signaling pathway
			mmu-miR-1933-3p	Dap	Autophagy
			mmu-miR-770-5p	Rnf152	Autophagy mTOR signaling pathway
circRNA.5562	9.53	Up	mmu-miR-3100-5p	Bmp2	Hippo signaling pathway
mmu_circ_0005739	9.07	Up	mmu-miR-1897-5p	Map3k12	MAPK signaling pathway
			mmu-miR-3091-5p	Mef2c	MAPK signaling pathway
			mmu-miR-345-3p	Adrb2	cAMP signaling pathway Calcium signaling pathway
circRNA.5566	8.71	Up	mmu-miR-1953	Bmp2	Hippo signaling pathway
			mmu-miR-5114	Gli2	Hippo signaling pathway
			mmu-miR-3100-5p	Bmp2	Hippo signaling pathway
			mmu-miR-721	Map3k12	MAPK signaling pathway
			mmu-miR-1933-3p	Dap	Autophagy
circRNA.5563	8.47	Up	mmu-miR-105	Hspa1b	MAPK signaling pathway
			mmu-miR-5130	Orai2	Calcium signaling pathway
			mmu-miR-711	Slc7a5	mTOR signaling pathway
circRNA.27348	8.18	Up	mmu-miR-763	Tead3	Hippo signaling pathway
			mmu-miR-3104-5p	Fzd7	mTOR signaling pathway Hippo signaling pathway
circRNA.5573	7.95	Up	mmu-miR-1198-5p	Nr4a1	PI3K-Akt signaling pathway MAPK signaling pathway
			mmu-miR-1896	Bmp2	Hippo signaling pathway
			mmu-miR-1941-5p	Fzd7	mTOR signaling pathway Hippo signaling pathway
			mmu-miR-1954	Il1r1	MAPK signaling pathway
circRNA.27394	7.14	Up	mmu-miR-1904	Bnip3	autophagy
			mmu-miR-693-3p	Fgf11	PI3K-Akt signaling pathway MAPK signaling pathway
			mmu-miR-3097-3p	Ddit4	PI3K-Akt signaling pathway mTOR signaling pathway
			mmu-miR-199a-5p	Serpine1	HIF-1 signaling pathway Hippo signaling pathway
			mmu-miR-345-3p	Adrb2	cAMP signaling pathway Calcium signaling pathway

## Discussion

In the present study, a mouse model of sepsis-induced cardiomyopathy was established. A total of 1735 differently expressed circRNAs were identified in the LPS-treated mouse hearts. And 187 circRNAs were found to be related to 309 autophagy-associated mRNAs in septic myocardial tissue. Among these, 179 circRNAs were predicted to function as “miRNA sponges”.

There is a high mortality rate of SICM^2^. Dysregulated autophagy is one of the main pathophysiological events in SICM^25,26^. Some studies have found that circRNAs, such as circCDYL, circCUL2, circRNA_002581, and circEXOC5, were involved in regulating autophagy, even in an LPS-challenged mouse cell model^18,27–29^. The abovementioned reports suggest a role of circRNAs in sepsis-induced autophagy. In our study, we established an SICM mouse model and confirmed the occurrence of the autophagic response in the mouse myocardium tissue by testing the protein levels and performing GSEA for LC3 and p62. In total, 1735 dysregulated circRNAs (including 990 upregulated and 745 downregulated circRNAs) were found in septic mice myocardium tissues using microarrays. In these DE circRNAs, 187 circRNAs are related to autophagy on GO and KEGG pathway analyses.

Autophagy is a fundamental cell protection pathway, and lysosomes are the site of intracellular autophagy^30–32^. As autophagy-marker proteins, LC3 and p62 are related to autophagosomal membrane formation^33^. In the present study, the p62 protein expression decreased, while the ratio of LC3 II to LC3 I increased after LPS treatment (*vs.* control group, *p* < 0.05). This is consistent with the findings from previous studies^34,35^. Although autophagy was not present in the top 30 pathways shown in Fig. 3A, B, we still found that the gene set related to “positive regulation of autophagy” was enriched in the LPS group by GSEA.

Several molecular mechanisms may participate in autophagy. In our study, we found nine autophagy-associated endogenous signaling pathways based on KEGG pathway analysis. These signaling pathways associated with autophagy are involved in various cellular and animal models^36–39^. For example, ER stress-induced autophagy, which was mediated by oxidative stress, decreased via the modulation of the PI3K-related cascade reaction in acute lung injury in LPS-induced mice^40^. In an ischemic/reperfusion-induced H9C2 cell injury model, autophagy induced by HIF-1α/BNIP3 signaling pathway protects the myocardium^41^. Laminar flow-induced endothelial autophagy and SIRT1 expression due to inhibited Hippo/YAP signaling pathways interrupt atherosclerotic plaque formation^42^. Notably, the HIF-1 and Hippo signaling pathways are involved in autophagy regulation, but the regulatory role has not been adequately explored in SICM-induced autophagy.

The role of circRNAs in SICM-induced autophagy has not been fully explored. Additionally, circRNAs that act as “sponges” are involved in the regulation of target gene expression. Depending on GO and KEGG analyses, we found 179 autophagy-related circRNAs that may bind to miRNAs. Autophagy-associated mRNA-binding sites also exist on the same miRNAs. Thus, we predicted the SICM-induced autophagy-related target genes using the ceRNA networks. For example, circRNA.27393 showed the top fold-change among the autophagy-related circRNAs and might regulate the mRNAs, such as the death-associated protein (DAP), ring-finger protein 152 (rnf152), and Ajuba by sponging mmu-miR-1933-3p, mmu-miR-448-5p, mmu-miR-125a-5p, and mmu-miR-125b-5p. These predicted mRNAs are associated with autophagy^43–45^. Other predicted autophagy-related mRNAs identified on ceRNA analysis in our research have also been proved to be related to autophagy, such as Bnip3, PPP2R2A, eEF2K, and IGF1^46–49^.

In our previous research, we identified the mitochondrial function-associated lncRNAs in SICM^19^. Bnip3 and PPP2R2A are predicted to be autophagy-related mRNAs and are associated with mitophagy^46,47,50^. These results showed that circRNAs and lncRNAs might regulate mitochondrial function and degradation after SICM. Ajuba Tead3, Serpine1, Gli2, and Bmp2 were predicted to be related to the Hippo signaling pathway and were involved in autophagy^45,51–54^. Serpine1 and Bcl2 were predicted to be related to the HIF signaling pathway and autophagy^51,55^. Therefore, our results provide new ideas to further evaluate the role of the HIF-1 and Hippo signaling pathways in LPS-induced cardiomyocyte autophagy. The role of certain predicted circRNAs and mRNAs in SICM-induced autophagy has not been elucidated and should be evaluated in future studies.

Sepsis is a life-threatening organ dysfunction. Autophagy is a major pathogenesis of sepsis-induced cardiomyopathy. Our study identified differently expressed circRNAs in the hearts of septic mice. We also gained some specific circRNAs and their potential target mRNAs which might be involved in autophagy in septic hearts. These findings offer a fine view of circRNAs and might allow developing new treatment strategies for sepsis-induced cardiomyopathy and reducing the incidence and mortality of sepsis.

Because of the limitations of our detection methods, our experiment also has some limitations. First, the present study only focused on the acute phase of sepsis. Expression profiles of cirRNAs associated with autophagy in the chronic phase of sepsis needs to be further explored, which might provide a more panoramic view of autophagy-related circRNAs in sepsis-induced cardiomyopathy. Second, the combination of various methods is more reliable to detect circRNA, such as PCR, RNase R, and Northern blot. We explored the circRNAs using only qRT-PCR^56,57^. Third, autophagy-related pathways were not studied in detail, and we only screened circRNAs based on the reported autophagy pathways. Therefore, some potentially undiscovered circRNAs may be missed. Fourth, although we identified some potential autophagy-related circRNAs (such as circRNA.27393, circRNA.27392, and circRNA.5564), the mechanism of action of these circRNAs in SICM needs further in vivo and in vitro studies.

## Conclusions

Our data indicate that the circRNAs, including circRNA.27393, may influence SICM-induced autophagy. Our research provides a new potential treatment strategy for SICM via the regulation of autophagy by circRNAs.

## Supplementary Information

Supplementary Information.

## Data Availability

The microarray data of circRNAs and mRNAs have been deposited in the GEO database (GSE142615).
