# Supplementary material for: Identification of autophagy-associated circRNAs in sepsis-induced cardiomyopathy of mice
Source: Sci Rep. 2023 Jul 21;13:11807. doi: 10.1038/s41598-023-38998-7 (PMC10361974; doi:10.1038/s41598-023-38998-7)
Supplement: Supplementary file 1 — Supplementary Information. [file 41598_2023_38998_MOESM1_ESM.pdf]

# Supplementary figure legends

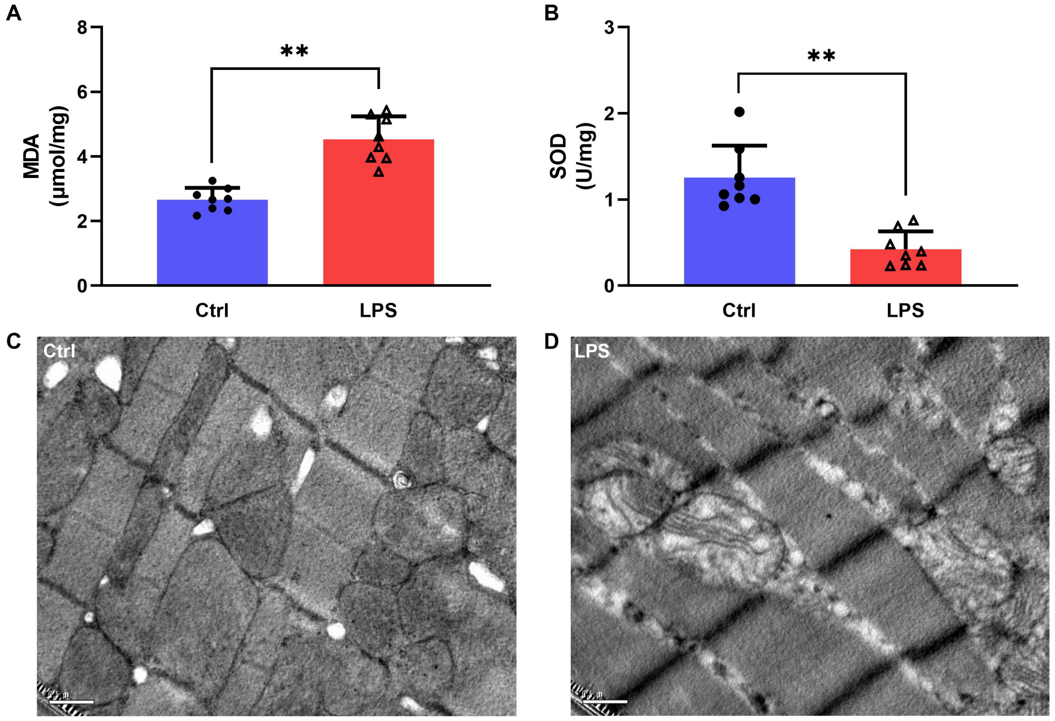


**Figure S1 MDA content, SOD activity, and mitochondrial morphology in septic mice hearts.** (A-B) MDA content and SOD activity. ***p* < 0.01 vs Ctrl group. Data are presented as mean ± SD, n = 8. (C-D) Representative ventricular myocardial ultrastructure (bar = 0.5 μm, Original magnification: 15,000)

# Supplmentary Tables

**Table S1 All the differently expressed mRNAs in autophagy-related signaling pathways**

| Serial number | GeneSymbol | Type | pvalues | foldchange | Regulation | Chromosome |
|---|---|---|---|---|---|---|
| 1 | 1300017J02Rik | mRNA | 0.000694138 | 2.278171768 | up | chr9 |
| 2 | ACP5 | mRNA | 1.85032E-05 | 7.747118119 | up | chr9 |
| 3 | ADCY3 | mRNA | 7.60916E-05 | 0.358576526 | down | chr12 |
| 4 | ADCY7 | mRNA | 2.94328E-05 | 0.191418936 | down | chr8 |
| 5 | Adcy8 | mRNA | 0.008220069 | 0.335743819 | down | chr15 |
| 6 | ADORA2B | mRNA | 9.68829E-07 | 17.30744257 | up | chr11 |
| 7 | Adrb2 | mRNA | 0.001205978 | 0.457127674 | down | chr18 |
| 8 | AGTR1A | mRNA | 0.001118932 | 0.445425726 | down | chr13 |
| 9 | Ajuba | mRNA | 0.000557181 | 0.303107473 | down | chr14 |
| 10 | ANGPT2 | mRNA | 1.89827E-06 | 3.310548682 | up | chr8 |
| 11 | Apc2 | mRNA | 9.28163E-06 | 0.451252368 | down | chr10 |
| 12 | ARRB1 | mRNA | 0.000169292 | 0.434216205 | down | chr7 |
| 13 | ARRB2 | mRNA | 2.75094E-05 | 0.342495896 | down | chr11 |
| 14 | Arsb | mRNA | 0.000141238 | 0.403973227 | down | chr13 |
| 15 | ATF4 | mRNA | 0.000111555 | 2.885630584 | up | chr15 |
| 16 | ATP1B2 | mRNA | 3.58075E-06 | 0.405902697 | down | chr11 |
| 17 | Atp2b4 | mRNA | 7.0018E-06 | 2.891682184 | up | chr1 |
| 18 | Atp6v0d2 | mRNA | 8.13848E-05 | 0.150516295 | down | chr4 |
| 19 | Avpr1a | mRNA | 0.008608425 | 2.175378742 | up | chr10 |
| 20 | Axin2 | mRNA | 0.029877704 | 0.477048413 | down | chr11 |
| 21 | Bad | mRNA | 0.000805594 | 0.483028107 | down | chr19 |
| 22 | Bcl2 | mRNA | 2.20877E-05 | 0.277421424 | down | chr1 |
| 23 | BCL2L1 | mRNA | 3.8517E-06 | 4.126246694 | up | chr2 |
| 24 | BDNF | mRNA | 0.000228824 | 0.411174489 | down | chr2 |
| 25 | BIRC2 | mRNA | 1.9616E-06 | 3.325580246 | up | chr9 |
| 26 | Bmp2 | mRNA | 0.000243396 | 3.295868914 | up | chr2 |
| 27 | Bmp7 | mRNA | 0.000394583 | 0.391900156 | down | chr2 |
| 28 | Bmp8a | mRNA | 0.002184691 | 7.460834794 | up | chr4 |
| 29 | Cacna1d | mRNA | 0.00279454 | 2.004720701 | up | chr14 |
| 30 | CACNA1G | mRNA | 0.00078614 | 0.438156617 | down | chr11 |
| 31 | Cacna1h | mRNA | 0.001244428 | 0.426061943 | down | chr17 |
| 32 | Cacna2d2 | mRNA | 0.01577159 | 3.239181466 | up | chr9 |
| 33 | Cacnb1 | mRNA | 1.90075E-05 | 0.484684485 | down | chr11 |
| 34 | CACNB4 | mRNA | 0.009358815 | 0.394631016 | down | chr2 |
| 35 | Cacng1 | mRNA | 0.000451203 | 5.123487462 | up | chr11 |
| 36 | CALML4 | mRNA | 0.000522523 | 0.159465489 | down | chr9 |
| 37 | Camk2a | mRNA | 0.007915295 | 0.453868853 | down | chr18 |
| 38 | CD14 | mRNA | 3.33941E-07 | 4.874737367 | up | chr18 |
| 39 | CDC25B | mRNA | 0.000237991 | 0.31803019 | down | chr2 |
| 40 | CDKN1A | mRNA | 4.52026E-08 | 26.02209145 | up | chr17 |
| 41 | Cnga3 | mRNA | 0.020920823 | 0.325661377 | down | chr1 |
| 42 | Cngb1 | mRNA | 0.004128493 | 0.144760312 | down | chr8 |
| 43 | COL1A1 | mRNA | 0.003476636 | 0.397087736 | down | chr11 |
| 44 | COL1A2 | mRNA | 0.000144042 | 0.349181922 | down | chr6 |

| | | | | | | |
|---|---|---|---|---|---|---|
| 45 | Col2a1 | mRNA | 0.004772727 | 0.405261168 | down | chr15 |
| 46 | COL4A3 | mRNA | 0.001964998 | 0.383143202 | down | chr1 |
| 47 | COL4A6 | mRNA | 6.35861E-05 | 0.369265801 | down | chrX |
| 48 | COL6A1 | mRNA | 0.000314285 | 0.436793517 | down | chr10 |
| 49 | COL6A2 | mRNA | 0.000854589 | 0.495789481 | down | chr10 |
| 50 | COL6A3 | mRNA | 0.005635847 | 0.361247409 | down | chr1 |
| 51 | COL6A6 | mRNA | 0.000952647 | 0.259049663 | down | chr9 |
| 52 | Col9a3 | mRNA | 0.015674849 | 4.074811817 | up | chr2 |
| 53 | Comp | mRNA | 0.003570413 | 0.413233468 | down | chr8 |
| 54 | Creb3l1 | mRNA | 7.03976E-06 | 2.920167956 | up | chr2 |
| 55 | CSF1 | mRNA | 2.69162E-05 | 5.546060559 | up | chr3 |
| 56 | CSF1R | mRNA | 4.93537E-05 | 0.466818176 | down | chr18 |
| 57 | CSF3 | mRNA | 4.25203E-07 | 399.336338 | up | chr11 |
| 58 | Csf3r | mRNA | 0.000939185 | 23.37031294 | up | chr4 |
| 59 | Csnk1e | mRNA | 0.008258383 | 0.431074526 | down | chr15 |
| 60 | Ctsg | mRNA | 0.001392579 | 4.0718868 | up | chr14 |
| 61 | CTSK | mRNA | 0.003116643 | 0.408243426 | down | chr3 |
| 62 | CYBB | mRNA | 1.19361E-07 | 4.751480395 | up | chrX |
| 63 | CYSLTR1 | mRNA | 5.18842E-05 | 0.351168468 | down | chrX |
| 64 | Cysltr2 | mRNA | 0.000648165 | 2.14749799 | up | chr14 |
| 65 | DAXX | mRNA | 2.34968E-05 | 4.227897584 | up | chr17 |
| 66 | DDIT4 | mRNA | 5.27463E-05 | 6.097113588 | up | chr10 |
| 67 | DLG2 | mRNA | 0.000609937 | 0.173519578 | down | chr7 |
| 68 | DUSP10 | mRNA | 0.00037655 | 0.404933352 | down | chr1 |
| 69 | DUSP2 | mRNA | 1.12667E-06 | 22.41912742 | up | chr2 |
| 70 | Dusp3 | mRNA | 1.06945E-06 | 0.495683729 | down | chr11 |
| 71 | DUSP6 | mRNA | 0.000216723 | 0.467101964 | down | chr10 |
| 72 | DVL2 | mRNA | 0.002613606 | 0.491617383 | down | chr11 |
| 73 | EDN1 | mRNA | 0.001823453 | 2.110331567 | up | chr13 |
| 74 | EEF2K | mRNA | 0.000102936 | 2.172969578 | up | chr7 |
| 75 | EFNA1 | mRNA | 0.000118367 | 0.268873839 | down | chr3 |
| 76 | Efna5 | mRNA | 0.005403657 | 2.076107915 | up | chr17 |
| 77 | EGFR | mRNA | 1.27584E-05 | 3.778491303 | up | chr11 |
| 78 | ENO2 | mRNA | 0.000544909 | 3.45667165 | up | chr6 |
| 79 | EPOR | mRNA | 9.59471E-05 | 0.219866515 | down | chr9 |
| 80 | FAS | mRNA | 1.25117E-08 | 5.67809294 | up | chr19 |
| 81 | Fbp1 | mRNA | 0.005924256 | 2.068668169 | up | chr13 |
| 82 | Fgf11 | mRNA | 1.49773E-05 | 0.432545257 | down | chr11 |
| 83 | Fgf14 | mRNA | 0.017975033 | 0.465016971 | down | chr14 |
| 84 | FGF16 | mRNA | 0.001561015 | 0.359006212 | down | chrX |
| 85 | FGF2 | mRNA | 0.001948718 | 4.541277607 | up | chr3 |
| 86 | FGF23 | mRNA | 8.79869E-05 | 27.25024804 | up | chr6 |
| 87 | Fgf6 | mRNA | 0.000379841 | 0.30978487 | down | chr6 |
| 88 | Fgf7 | mRNA | 0.000261453 | 0.459854563 | down | chr2 |
| 89 | FGF9 | mRNA | 1.78742E-05 | 0.425041156 | down | chr14 |
| 90 | FGFR3 | mRNA | 0.002221877 | 0.471231148 | down | chr5 |
| 91 | FLNB | mRNA | 2.30244E-05 | 5.023005042 | up | chr14 |
| 92 | FLT1 | mRNA | 2.21441E-06 | 0.420555563 | down | chr5 |
| 93 | FOS | mRNA | 0.000496179 | 6.601160505 | up | chr12 |
| 94 | FRMD6 | mRNA | 0.00079592 | 0.490162572 | down | chr12 |

| | | | | | | |
|---|---|---|---|---|---|---|
| 95 | FXYD2 | mRNA | 0.000146014 | 0.324312911 | down | chr9 |
| 96 | FZD2 | mRNA | 4.95103E-05 | 0.342721777 | down | chr11 |
| 97 | FZD7 | mRNA | 0.002741999 | 0.475637201 | down | chr1 |
| 98 | Gabbr1 | mRNA | 0.001211896 | 0.444963961 | down | chr17 |
| 99 | GADD45B | mRNA | 0.000248349 | 6.72942603 | up | chr10 |
| 100 | GADD45G | mRNA | 0.003084402 | 3.553008431 | up | chr13 |
| 101 | Gga2 | mRNA | 0.001508428 | 0.272255213 | down | chr7 |
| 102 | GIPR | mRNA | 0.01161097 | 0.399243971 | down | chr7 |
| 103 | Gli1 | mRNA | 9.7989E-08 | 0.176880299 | down | chr10 |
| 104 | Gli2 | mRNA | 7.51343E-08 | 0.105958635 | down | chr1 |
| 105 | Gm2a | mRNA | 0.000715752 | 0.416674547 | down | chr11 |
| 106 | GNG11 | mRNA | 2.53715E-10 | 0.203249155 | down | chr6 |
| 107 | GNG12 | mRNA | 1.01288E-06 | 2.311679742 | up | chr6 |
| 108 | Gng2 | mRNA | 0.002981945 | 0.415935533 | down | chr14 |
| 109 | Gng8 | mRNA | 0.000209382 | 0.115337403 | down | chr7 |
| 110 | Gria3 | mRNA | 0.000482538 | 0.308614258 | down | chrX |
| 111 | Gusb | mRNA | 4.87727E-05 | 0.494425507 | down | chr5 |
| 112 | Hcar2 | mRNA | 0.000539369 | 6.400076631 | up | chr5 |
| 113 | Hexb | mRNA | 7.0752E-05 | 0.377125187 | down | chr13 |
| 114 | Hif1a | mRNA | 0.000893749 | 2.353835104 | up | chr12 |
| 115 | Hmgcr | mRNA | 0.000348911 | 2.068497051 | up | chr13 |
| 116 | HMOX1 | mRNA | 0.000443665 | 3.554023651 | up | chr8 |
| 117 | HSPA1A | mRNA | 0.000640983 | 2.49435454 | up | chr17 |
| 118 | Hspa1b | mRNA | 0.000303911 | 2.364615859 | up | chr17 |
| 119 | Htr7 | mRNA | 9.70814E-06 | 0.224125263 | down | chr19 |
| 120 | HYAL1 | mRNA | 0.001515981 | 0.499176226 | down | chr9 |
| 121 | Ids | mRNA | 1.8514E-05 | 0.460492246 | down | chrX |
| 122 | IFNAR2 | mRNA | 6.57034E-06 | 2.908137289 | up | chr16 |
| 123 | IFNG | mRNA | 2.10924E-07 | 7.636440632 | up | chr10 |
| 124 | IFNGR1 | mRNA | 2.5292E-07 | 2.414373338 | up | chr10 |
| 125 | IGF1 | mRNA | 6.80069E-05 | 0.31444864 | down | chr10 |
| 126 | IL1A | mRNA | 0.000965517 | 12.84639119 | up | chr2 |
| 127 | IL1B | mRNA | 3.9849E-05 | 39.37285401 | up | chr2 |
| 128 | IL1R1 | mRNA | 1.21621E-05 | 2.441897383 | up | chr1 |
| 129 | IL1R2 | mRNA | 1.68683E-06 | 69.16418251 | up | chr1 |
| 130 | Il2rb | mRNA | 0.015537879 | 2.513214707 | up | chr15 |
| 131 | Il2rg | mRNA | 3.06963E-05 | 5.642949004 | up | chrX |
| 132 | Il3ra | mRNA | 0.000432124 | 2.717363985 | up | chr14 |
| 133 | IL4RA | mRNA | 1.76373E-05 | 8.21361563 | up | chr7 |
| 134 | IL6 | mRNA | 1.36451E-05 | 860.7737718 | up | chr5 |
| 135 | Il6ra | mRNA | 0.006440644 | 2.008414596 | up | chr3 |
| 136 | ITGA11 | mRNA | 0.00016672 | 0.133581592 | down | chr9 |
| 137 | Itga2 | mRNA | 0.024636524 | 2.458944491 | up | chr13 |
| 138 | Itga2b | mRNA | 0.000253033 | 2.49901303 | up | chr11 |
| 139 | ITGA6 | mRNA | 3.37768E-05 | 0.15528509 | down | chr2 |
| 140 | ITGA8 | mRNA | 0.004098176 | 0.15849984 | down | chr2 |
| 141 | ITGB3 | mRNA | 5.43865E-05 | 4.070432971 | up | chr11 |
| 142 | ITGB4 | mRNA | 1.36516E-06 | 0.403784384 | down | chr11 |
| 143 | ITGB6 | mRNA | 0.001004215 | 0.420815313 | down | chr2 |
| 144 | ITPKA | mRNA | 1.3148E-06 | 3.583249244 | up | chr2 |

| | | | | | | |
|---|---|---|---|---|---|---|
| 145 | ITPKC | mRNA | 0.003469736 | 3.484312785 | up | chr7 |
| 146 | ITPR3 | mRNA | 0.001445636 | 0.368859137 | down | chr17 |
| 147 | JAK2 | mRNA | 3.17787E-06 | 3.042589295 | up | chr19 |
| 148 | KDR | mRNA | 0.001447836 | 0.171850169 | down | chr5 |
| 149 | KITL | mRNA | 0.001150583 | 0.427466506 | down | chr10 |
| 150 | LAMA3 | mRNA | 0.000112837 | 0.357608451 | down | chr18 |
| 151 | LAMB1 | mRNA | 0.000245715 | 0.495386352 | down | chr12 |
| 152 | LAMB3 | mRNA | 0.010001044 | 0.398275386 | down | chr1 |
| 153 | LAMC2 | mRNA | 8.6182E-06 | 2.947862779 | up | chr1 |
| 154 | LAPTM5 | mRNA | 0.002624621 | 0.458295032 | down | chr4 |
| 155 | LEPR | mRNA | 0.000130477 | 0.326350138 | down | chr4 |
| 156 | LPAR3 | mRNA | 0.002109163 | 0.333110574 | down | chr3 |
| 157 | Lpar4 | mRNA | 0.000124536 | 0.305566059 | down | chrX |
| 158 | Lpar6 | mRNA | 0.000102364 | 0.469353782 | down | chr14 |
| 159 | MANBA | mRNA | 5.40926E-07 | 0.470187998 | down | chr3 |
| 160 | Map2k6 | mRNA | 2.78357E-05 | 0.329017738 | down | chr11 |
| 161 | MAP3K12 | mRNA | 0.000108575 | 0.43979166 | down | chr15 |
| 162 | MAP3K6 | mRNA | 0.000611289 | 9.404143099 | up | chr4 |
| 163 | Map3k8 | mRNA | 7.37286E-07 | 6.851998081 | up | chr18 |
| 164 | Map4k2 | mRNA | 0.016159134 | 0.395501624 | down | chr19 |
| 165 | MAP4K3 | mRNA | 0.002597804 | 0.498400219 | down | chr17 |
| 166 | MAPK12 | mRNA | 5.27788E-06 | 0.43508515 | down | chr15 |
| 167 | MAPK3 | mRNA | 0.000145922 | 0.34341741 | down | chr7 |
| 168 | Mapk8ip1 | mRNA | 0.002362234 | 0.482053984 | down | chr2 |
| 169 | MECOM | mRNA | 0.000638045 | 0.360438231 | down | chr3 |
| 170 | MEF2C | mRNA | 0.001010881 | 0.498928974 | down | chr13 |
| 171 | MET | mRNA | 0.003184349 | 4.851655212 | up | chr6 |
| 172 | Mras | mRNA | 0.015054552 | 0.46646517 | down | chr9 |
| 173 | MYC | mRNA | 5.34854E-06 | 12.48939751 | up | chr15 |
| 174 | Naglu | mRNA | 9.75919E-06 | 0.482025873 | down | chr11 |
| 175 | NAPSA | mRNA | 0.001732004 | 4.096309633 | up | chr7 |
| 176 | Nfatc1 | mRNA | 0.000717743 | 0.495550344 | down | chr18 |
| 177 | NFKB2 | mRNA | 3.71396E-06 | 4.051804312 | up | chr19 |
| 178 | NFKBIA | mRNA | 1.10502E-07 | 6.77014079 | up | chr12 |
| 179 | Ngf | mRNA | 0.002781119 | 2.087504975 | up | chr3 |
| 180 | Nkd1 | mRNA | 0.00109117 | 0.269835744 | down | chr8 |
| 181 | NOS3 | mRNA | 0.007851568 | 0.367736418 | down | chr5 |
| 182 | NPR1 | mRNA | 0.000270759 | 0.368530278 | down | chr3 |
| 183 | Nr4a1 | mRNA | 0.007653154 | 2.293307464 | up | chr15 |
| 184 | NTF3 | mRNA | 0.000209236 | 0.108337692 | down | chr6 |
| 185 | Ntrk2 | mRNA | 6.64623E-05 | 3.02285355 | up | chr13 |
| 186 | Orai2 | mRNA | 0.000544563 | 3.055900619 | up | chr5 |
| 187 | Osm | mRNA | 0.001178247 | 2.001274754 | up | chr11 |
| 188 | OSMR | mRNA | 3.89697E-06 | 8.638079516 | up | chr15 |
| 189 | P2RX6 | mRNA | 0.01717217 | 0.381526267 | down | chr16 |
| 190 | P2RX7 | mRNA | 0.00133748 | 0.244812272 | down | chr5 |
| 191 | Pard6g | mRNA | 0.000232444 | 0.470459835 | down | chr18 |
| 192 | PDE4B | mRNA | 0.001520485 | 4.919365297 | up | chr4 |
| 193 | PDGFB | mRNA | 6.28187E-05 | 0.494730012 | down | chr15 |
| 194 | Pdgfc | mRNA | 0.004195509 | 0.450822602 | down | chr3 |

| | | | | | | |
|---|---|---|---|---|---|---|
| 195 | Pdgfra | mRNA | 5.62506E-05 | 0.461412449 | down | chr5 |
| 196 | PFKFB2 | mRNA | 0.000321913 | 0.431801134 | down | chr1 |
| 197 | PFKFB3 | mRNA | 7.03519E-05 | 30.46002281 | up | chr2 |
| 198 | PGF | mRNA | 1.3336E-07 | 15.41550099 | up | chr12 |
| 199 | PHLPP1 | mRNA | 5.62367E-07 | 0.39549399 | down | chr1 |
| 200 | PIK3AP1 | mRNA | 0.000224444 | 5.451187327 | up | chr19 |
| 201 | Pik3cg | mRNA | 0.00030097 | 0.400274589 | down | chr12 |
| 202 | PIK3R1 | mRNA | 0.000270217 | 3.098697911 | up | chr13 |
| 203 | Pik3r2 | mRNA | 0.000520115 | 0.440558522 | down | chr8 |
| 204 | PIK3R5 | mRNA | 5.83856E-06 | 41.72424786 | up | chr11 |
| 205 | Pik3r6 | mRNA | 9.52799E-07 | 2.055388953 | up | chr11 |
| 206 | PKN3 | mRNA | 0.017654525 | 0.282076623 | down | chr2 |
| 207 | PLA2G15 | mRNA | 0.00106976 | 0.354932756 | down | chr8 |
| 208 | PLA2G4D | mRNA | 0.005143591 | 2.331140595 | up | chr2 |
| 209 | PLCB1 | mRNA | 0.001028071 | 0.347801049 | down | chr2 |
| 210 | Pld2 | mRNA | 0.000246105 | 0.494772672 | down | chr11 |
| 211 | Ppp2r2a | mRNA | 0.00318088 | 2.044459973 | up | chr14 |
| 212 | Prkca | mRNA | 0.000190553 | 0.379819885 | down | chr11 |
| 213 | Prkcb | mRNA | 0.000660716 | 0.173874751 | down | chr7 |
| 214 | Prkcz | mRNA | 0.000112595 | 0.486795253 | down | chr4 |
| 215 | Ptafr | mRNA | 5.64694E-06 | 4.519823125 | up | chr4 |
| 216 | PTPRR | mRNA | 0.000467278 | 0.365150368 | down | chr10 |
| 217 | RAC2 | mRNA | 0.000516125 | 2.797175693 | up | chr15 |
| 218 | Rac3 | mRNA | 5.06077E-05 | 2.022635247 | up | chr11 |
| 219 | Rapgef4 | mRNA | 0.001090939 | 0.406046606 | down | chr2 |
| 220 | Rasgrf1 | mRNA | 0.003681023 | 6.057380248 | up | chr9 |
| 221 | RASGRF2 | mRNA | 0.000802192 | 0.111602191 | down | chr13 |
| 222 | RASGRP1 | mRNA | 1.91496E-05 | 3.951169845 | up | chr2 |
| 223 | RASGRP2 | mRNA | 0.004569591 | 0.205998816 | down | chr19 |
| 224 | RASGRP3 | mRNA | 9.30193E-07 | 0.20989857 | down | chr17 |
| 225 | RASGRP4 | mRNA | 0.000386847 | 0.492469581 | down | chr7 |
| 226 | RELA | mRNA | 0.000140014 | 2.474552493 | up | chr19 |
| 227 | RELB | mRNA | 3.98617E-05 | 4.36670491 | up | chr7 |
| 228 | RNF152 | mRNA | 0.001902641 | 0.246726599 | down | chr1 |
| 229 | RPS6KA1 | mRNA | 0.000423079 | 0.462572248 | down | chr4 |
| 230 | Sav1 | mRNA | 4.1744E-05 | 2.301668763 | up | chr12 |
| 231 | Scd1 | mRNA | 2.67597E-05 | 0.402834878 | down | chr19 |
| 232 | SCD2 | mRNA | 0.000244683 | 0.26138159 | down | chr19 |
| 233 | SCD4 | mRNA | 0.000415159 | 2.003502608 | up | chr19 |
| 234 | SERPINE1 | mRNA | 1.04829E-05 | 6.48656863 | up | chr5 |
| 235 | Sesn2 | mRNA | 0.018024139 | 2.243703422 | up | chr4 |
| 236 | SGSH | mRNA | 3.90914E-05 | 0.46847984 | down | chr11 |
| 237 | SLC17A5 | mRNA | 1.32675E-05 | 0.484077246 | down | chr9 |
| 238 | Slc38a9 | mRNA | 0.002334562 | 0.468795731 | down | chr13 |
| 239 | SLC3A2 | mRNA | 2.02716E-06 | 2.869941097 | up | chr19 |
| 240 | SLC7A5 | mRNA | 2.30632E-05 | 4.836835437 | up | chr8 |
| 241 | SMAD1 | mRNA | 0.00437548 | 2.354131783 | up | chr8 |
| 242 | SNAI2 | mRNA | 3.14809E-06 | 0.22885244 | down | chr16 |
| 243 | Sort1 | mRNA | 0.000341007 | 0.482630392 | down | chr3 |
| 244 | SOX9 | mRNA | 0.005935163 | 0.449423156 | down | chr11 |

| | | | | | | |
|---|---|---|---|---|---|---|
| 245 | SPHK1 | mRNA | 2.00664E-07 | 31.90160412 | up | chr11 |
| 246 | STAT3 | mRNA | 1.54468E-05 | 2.545375853 | up | chr11 |
| 247 | Stim2 | mRNA | 0.000173226 | 0.463099234 | down | chr5 |
| 248 | SUCNR1 | mRNA | 0.000144042 | 0.106868482 | down | chr3 |
| 249 | SYK | mRNA | 2.57161E-05 | 3.226913899 | up | chr13 |
| 250 | Tbc1d1 | mRNA | 0.000322979 | 2.412823516 | up | chr5 |
| 251 | TBXA2R | mRNA | 3.02155E-05 | 0.164275812 | down | chr10 |
| 252 | TCIRG1 | mRNA | 1.30164E-05 | 2.819100697 | up | chr19 |
| 253 | TEAD2 | mRNA | 1.04973E-05 | 0.45337945 | down | chr7 |
| 254 | TEAD3 | mRNA | 6.35463E-07 | 2.344526865 | up | chr17 |
| 255 | TEK | mRNA | 0.000103265 | 0.252509353 | down | chr4 |
| 256 | TGFB3 | mRNA | 1.14708E-05 | 0.410115625 | down | chr12 |
| 257 | THBS1 | mRNA | 0.001064683 | 5.965938034 | up | chr2 |
| 258 | Thbs2 | mRNA | 7.19793E-05 | 0.035228503 | down | chr17 |
| 259 | THBS4 | mRNA | 0.000757663 | 5.059769319 | up | chr13 |
| 260 | TIMP1 | mRNA | 7.57984E-08 | 26.01339947 | up | chrX |
| 261 | TLR2 | mRNA | 1.8212E-05 | 8.719396345 | up | chr3 |
| 262 | Tlr4 | mRNA | 0.000660791 | 2.003943252 | up | chr4 |
| 263 | TNC | mRNA | 0.003107687 | 3.040071146 | up | chr4 |
| 264 | TNF | mRNA | 2.31057E-05 | 11.8568212 | up | chr17 |
| 265 | TNFRSF1A | mRNA | 0.000500699 | 2.36986862 | up | chr6 |
| 266 | TNXB | mRNA | 3.13118E-05 | 0.395071199 | down | chr17 |
| 267 | TRF | mRNA | 5.77586E-06 | 0.461574609 | down | chr9 |
| 268 | VAV1 | mRNA | 0.000255462 | 3.07445114 | up | chr17 |
| 269 | VAV3 | mRNA | 6.23472E-07 | 0.316981693 | down | chr3 |
| 270 | Vegfa | mRNA | 0.000100786 | 0.489614652 | down | chr17 |
| 271 | VEGFC | mRNA | 7.35106E-05 | 0.345854594 | down | chr8 |
| 272 | VEGFD | mRNA | 0.000853802 | 0.42870015 | down | chrX |
| 273 | Vipr2 | mRNA | 0.000392337 | 0.144255892 | down | chr12 |
| 274 | VTN | mRNA | 0.000400872 | 0.362415575 | down | chr11 |
| 275 | Wnt2 | mRNA | 0.001880732 | 0.412232853 | down | chr6 |
| 276 | WNT4 | mRNA | 0.002154445 | 8.026105529 | up | chr4 |
| 277 | Wnt5b | mRNA | 2.95466E-07 | 0.310862658 | down | chr6 |
| 278 | Wnt6 | mRNA | 0.03240714 | 0.380676435 | down | chr1 |
| 279 | WNT9A | mRNA | 0.000463945 | 0.433204591 | down | chr11 |

**Table S2 All the differently expressed circRNAs in autophagy-related signaling pathways**

| Serial number | CircRNA_ID | Type | pvalues | Foldchange | Regulation | circ_chrom | Hostgene |
|---|---|---|---|---|---|---|---|
| 1 | circRNA.11009 | circRNA | 0.001053621 | 0.452389882 | down | chr11 | Col1a1 |
| 2 | circRNA.11010 | circRNA | 0.001131877 | 0.480234958 | down | chr11 | Col1a1 |
| 3 | circRNA.11013 | circRNA | 0.007621395 | 0.432053407 | down | chr11 | Col1a1 |
| 4 | circRNA.1255 | circRNA | 1.30276E-05 | 5.594952363 | up | chr18 | Map3k8 |
| 5 | circRNA.12832 | circRNA | 0.001480865 | 2.005143483 | up | chr11 | Egfr |
| 6 | circRNA.12833 | circRNA | 0.00862962 | 2.250441241 | up | chr11 | Egfr |
| 7 | circRNA.12834 | circRNA | 0.000160538 | 2.263616592 | up | chr11 | Egfr |
| 8 | circRNA.12835 | circRNA | 5.222E-07 | 2.780695918 | up | chr11 | Egfr |
| 9 | circRNA.12836 | circRNA | 9.26162E-06 | 2.562542184 | up | chr11 | Egfr |
| 10 | circRNA.12838 | circRNA | 0.00150193 | 2.134456089 | up | chr11 | Egfr |
| 11 | circRNA.12839 | circRNA | 0.000264697 | 2.150247089 | up | chr11 | Egfr |
| 12 | circRNA.12843 | circRNA | 2.43552E-05 | 2.928533307 | up | chr11 | Egfr |
| 13 | circRNA.12844 | circRNA | 0.003213364 | 2.810093875 | up | chr11 | Egfr |
| 14 | circRNA.13043 | circRNA | 6.92224E-05 | 2.270912816 | up | chr11 | Sphk1 |
| 15 | circRNA.13121 | circRNA | 7.57718E-05 | 0.310619918 | down | chr11 | Map2k6 |
| 16 | circRNA.13122 | circRNA | 0.000530375 | 0.335233513 | down | chr11 | Map2k6 |
| 17 | circRNA.13322 | circRNA | 0.04798225 | 2.691702596 | up | chr11 | Itgb3 |
| 18 | circRNA.13323 | circRNA | 0.000930612 | 2.848867785 | up | chr11 | Itgb3 |
| 19 | circRNA.13324 | circRNA | 0.000125091 | 4.415431919 | up | chr11 | Itgb3 |
| 20 | circRNA.13476 | circRNA | 2.11202E-05 | 3.235220544 | up | chr11 | Stat3 |
| 21 | circRNA.13478 | circRNA | 4.64414E-05 | 2.83225849 | up | chr11 | Stat3 |
| 22 | circRNA.13481 | circRNA | 0.000130296 | 3.329259568 | up | chr11 | Stat3 |
| 23 | circRNA.13749 | circRNA | 7.10782E-05 | 0.486231614 | down | chr10 | Igf1 |
| 24 | circRNA.13997 | circRNA | 6.2051E-06 | 0.473145352 | down | chr10 | Col6a1 |
| 25 | circRNA.14328 | circRNA | 0.000111414 | 3.892722631 | up | chr10 | Ddit4 |
| 26 | circRNA.15204 | circRNA | 0.011705335 | 0.221742981 | down | chr10 | Ptprr |
| 27 | circRNA.15366 | circRNA | 0.000155664 | 0.414737454 | down | chr10 | Kitl |
| 28 | circRNA.15367 | circRNA | 0.001296707 | 0.448901848 | down | chr10 | Kitl |
| 29 | circRNA.15513 | circRNA | 0.000153429 | 0.47427428 | down | chr1 | Col6a3 |
| 30 | circRNA.15514 | circRNA | 0.001103255 | 0.442182055 | down | chr1 | Col6a3 |
| 31 | circRNA.15516 | circRNA | 0.000743584 | 0.44778884 | down | chr1 | Col6a3 |
| 32 | circRNA.17736 | circRNA | 2.64762E-06 | 3.277908041 | up | chr1 | Atp2b4 |
| 33 | circRNA.18363 | circRNA | 2.84034E-05 | 0.485079547 | down | chrX | Ids |
| 34 | circRNA.18452 | circRNA | 9.64937E-06 | 2.37666495 | up | chrX | Timp1 |
| 35 | circRNA.18453 | circRNA | 3.73029E-05 | 2.674461162 | up | chrX | Timp1 |
| 36 | circRNA.1946 | circRNA | 0.000620939 | 0.454093989 | down | chr17 | Map4k3 |
| 37 | circRNA.1953 | circRNA | 0.002407971 | 0.494190526 | down | chr17 | Map4k3 |
| 38 | circRNA.19788 | circRNA | 1.37535E-05 | 0.164645979 | down | chr8 | Adcy7 |
| 39 | circRNA.19972 | circRNA | 1.23071E-06 | 0.353443373 | down | chr8 | Vegfc |
| 40 | circRNA.20257 | circRNA | 0.000271007 | 2.476654369 | up | chr7 | Dlg2 |
| 41 | circRNA.21541 | circRNA | 6.83307E-05 | 0.47798115 | down | chr5 | Flt1 |
| 42 | circRNA.21542 | circRNA | 0.001738668 | 0.380533108 | down | chr5 | Flt1 |
| 43 | circRNA.2387 | circRNA | 0.001071864 | 2.395763988 | up | chr17 | Efna5 |
| 44 | circRNA.24162 | circRNA | 0.000205665 | 0.457668418 | down | chr3 | Manba |
| 45 | circRNA.24164 | circRNA | 0.000505164 | 0.469138253 | down | chr3 | Manba |
| 46 | circRNA.24166 | circRNA | 5.17069E-05 | 0.462661468 | down | chr3 | Manba |
| 47 | circRNA.24390 | circRNA | 0.002969677 | 0.464088566 | down | chr3 | Vav3 |
| 48 | circRNA.24429 | circRNA | 2.10381E-05 | 6.275860791 | up | chr3 | Csf1 |
| 49 | circRNA.24430 | circRNA | 3.21236E-05 | 5.502126901 | up | chr3 | Csf1 |
| 50 | circRNA.24600 | circRNA | 0.004541738 | 2.604438581 | up | chr2 | Creb3l1 |
| 51 | circRNA.24601 | circRNA | 0.000188848 | 2.349539595 | up | chr2 | Creb3l1 |
| 52 | circRNA.24927 | circRNA | 0.003314941 | 0.200919678 | down | chr2 | Itga6 |
| 53 | circRNA.24929 | circRNA | 0.000351418 | 0.331008476 | down | chr2 | Itga6 |
| 54 | circRNA.25125 | circRNA | 0.002936208 | 0.429016876 | down | chr2 | Itgb6 |
| 55 | circRNA.25129 | circRNA | 0.00203312 | 0.468964405 | down | chr2 | Itgb6 |

| | | | | | | | |
|---|---|---|---|---|---|---|---|
| 56 | circRNA.25133 | circRNA | 0.001285103 | 0.347805182 | down | chr2 | Itgb6 |
| 57 | circRNA.26828 | circRNA | 0.000279382 | 0.399571084 | down | chr2 | Plcb1 |
| 58 | circRNA.27121 | circRNA | 0.000278271 | 0.215429079 | down | chr2 | Itga8 |
| 59 | circRNA.27348 | circRNA | 0.004261081 | 8.175117047 | up | chr2 | Thbs1 |
| 60 | circRNA.27349 | circRNA | 0.004210571 | 2.108062453 | up | chr2 | Thbs1 |
| 61 | circRNA.27351 | circRNA | 0.000437367 | 2.328466029 | up | chr2 | Thbs1 |
| 62 | circRNA.27392 | circRNA | 3.29012E-07 | 12.79919582 | up | chr2 | Pfkfb3 |
| 63 | circRNA.27393 | circRNA | 5.52283E-06 | 21.23872246 | up | chr2 | Pfkfb3 |
| 64 | circRNA.27394 | circRNA | 1.22084E-07 | 7.136921458 | up | chr2 | Pfkfb3 |
| 65 | circRNA.27630 | circRNA | 0.000366252 | 2.670787787 | up | chr19 | Slc3a2 |
| 66 | circRNA.27631 | circRNA | 0.007362063 | 2.204870165 | up | chr19 | Slc3a2 |
| 67 | circRNA.27819 | circRNA | 0.000536681 | 2.749574767 | up | chr19 | Rela |
| 68 | circRNA.27968 | circRNA | 1.00316E-06 | 2.621704012 | up | chr19 | Nfkb2 |
| 69 | circRNA.2818 | circRNA | 0.004813006 | 0.472764223 | down | chr17 | Tnxb |
| 70 | circRNA.2844 | circRNA | 3.84587E-05 | 4.132027996 | up | chr17 | Daxx |
| 71 | circRNA.2845 | circRNA | 0.000142259 | 4.196161722 | up | chr17 | Daxx |
| 72 | circRNA.3017 | circRNA | 0.002916199 | 0.453354432 | down | chr17 | Itpr3 |
| 73 | circRNA.3214 | circRNA | 0.003247997 | 0.452751748 | down | chr17 | Thbs2 |
| 74 | circRNA.322 | circRNA | 0.002594915 | 3.712426602 | up | chr19 | Jak2 |
| 75 | circRNA.5560 | circRNA | 0.000100269 | 3.09544988 | up | chr15 | Osmr |
| 76 | circRNA.5564 | circRNA | 0.000628902 | 10.76847746 | up | chr15 | Osmr |
| 77 | circRNA.5566 | circRNA | 3.23771E-06 | 8.707272797 | up | chr15 | Osmr |
| 78 | circRNA.6519 | circRNA | 1.54914E-05 | 3.908359736 | up | chr14 | Flnb |
| 79 | circRNA.6522 | circRNA | 1.11223E-05 | 4.223497413 | up | chr14 | Flnb |
| 80 | circRNA.6523 | circRNA | 5.74056E-06 | 3.427690953 | up | chr14 | Flnb |
| 81 | circRNA.6527 | circRNA | 3.71957E-06 | 2.599468016 | up | chr14 | Flnb |
| 82 | circRNA.6533 | circRNA | 0.000661292 | 2.939091937 | up | chr14 | Flnb |
| 83 | circRNA.6536 | circRNA | 0.006785795 | 2.705761008 | up | chr14 | Flnb |
| 84 | circRNA.6581 | circRNA | 0.00043951 | 4.203408981 | up | chr14 | Flnb |
| 85 | circRNA.6583 | circRNA | 8.21105E-05 | 2.178283139 | up | chr14 | Flnb |
| 86 | circRNA.6586 | circRNA | 1.37981E-06 | 3.867151577 | up | chr14 | Flnb |
| 87 | circRNA.6756 | circRNA | 0.001159814 | 2.160484474 | up | chr14 | Ppp2r2a |
| 88 | circRNA.6757 | circRNA | 0.001063196 | 2.232888318 | up | chr14 | Ppp2r2a |
| 89 | circRNA.6758 | circRNA | 0.000255368 | 2.249003256 | up | chr14 | Ppp2r2a |
| 90 | circRNA.7926 | circRNA | 0.000950812 | 0.399599599 | down | chr13 | Hexb |
| 91 | circRNA.8056 | circRNA | 0.003797045 | 0.371740907 | down | chr13 | Arsb |
| 92 | circRNA.8088 | circRNA | 0.00027301 | 0.109760411 | down | chr13 | Rasgrf2 |
| 93 | circRNA.8170 | circRNA | 0.00026631 | 0.468454063 | down | chr13 | Mef2c |
| 94 | circRNA.8376 | circRNA | 0.001608294 | 2.211191873 | up | chr13 | Ntrk2 |
| 95 | circRNA.8463 | circRNA | 0.000244065 | 2.518636233 | up | chr13 | Syk |
| 96 | circRNA.8464 | circRNA | 0.001378478 | 2.337143344 | up | chr13 | Syk |
| 97 | circRNA.8466 | circRNA | 0.001804056 | 4.317688928 | up | chr13 | Gadd45g |
| 98 | circRNA.9281 | circRNA | 0.001089198 | 2.630031617 | up | chr13 | Pik3r1 |
| 99 | circRNA.9282 | circRNA | 0.002355879 | 2.614414444 | up | chr13 | Pik3r1 |
| 100 | circRNA.9488 | circRNA | 0.001008524 | 3.026753852 | up | chr12 | Fos |
| 101 | circRNA.9764 | circRNA | 0.001698145 | 2.387371881 | up | chr12 | Hif1a |
| 102 | circRNA.9765 | circRNA | 0.00123259 | 2.267699884 | up | chr12 | Hif1a |
| 103 | circRNA.9796 | circRNA | 0.001169277 | 0.490266149 | down | chr12 | Frmd6 |
| 104 | circRNA.9809 | circRNA | 6.18738E-05 | 2.057132018 | up | chr12 | Sav1 |
| 105 | mmu_circ_0000079 | circRNA | 1.34915E-05 | 2.334737087 | up | chr1 | Atp2b4 |
| 106 | mmu_circ_0000837 | circRNA | 0.000126476 | 4.522719171 | up | chr18 | Map3k8 |
| 107 | mmu_circ_0001158 | circRNA | 1.32494E-05 | 0.33034386 | down | chr3 | Vav3 |
| 108 | mmu_circ_0002648 | circRNA | 4.09575E-05 | 0.472349674 | down | chr11 | Dusp3 |
| 109 | mmu_circ_0002762 | circRNA | 0.002749617 | 0.239503933 | down | chr11 | Map2k6 |
| 110 | mmu_circ_0002861 | circRNA | 0.009202445 | 3.645048181 | up | chr11 | Egfr |
| 111 | mmu_circ_0004176 | circRNA | 0.000424237 | 2.059978721 | up | chr12 | Hif1a |
| 112 | mmu_circ_0004400 | circRNA | 0.000637095 | 3.535061943 | up | chr13 | Pik3r1 |
| 113 | mmu_circ_0004829 | circRNA | 0.000446071 | 0.472547038 | down | chr13 | Mef2c |
| 114 | mmu_circ_0004830 | circRNA | 0.001073782 | 0.480439408 | down | chr13 | Mef2c |

| | | | | | | | |
|---|---|---|---|---|---|---|---|
| 115 | mmu_circ_0004873 | circRNA | 0.006395832 | 0.313690283 | down | chr13 | Rasgrf2 |
| 116 | mmu_circ_0004874 | circRNA | 0.0041232 | 0.400908757 | down | chr13 | Rasgrf2 |
| 117 | mmu_circ_0004891 | circRNA | 0.001061728 | 0.469845821 | down | chr13 | Arsb |
| 118 | mmu_circ_0005484 | circRNA | 3.1078E-05 | 3.108091803 | up | chr14 | Flnb |
| 119 | mmu_circ_0005852 | circRNA | 6.48978E-05 | 2.009153381 | up | chr15 | Atf4 |
| 120 | mmu_circ_0007014 | circRNA | 2.372E-06 | 0.237983843 | down | chr17 | Rasgrp3 |
| 121 | mmu_circ_0007907 | circRNA | 4.65809E-05 | 2.832508681 | up | chr19 | Rela |
| 122 | mmu_circ_0008005 | circRNA | 0.048441507 | 0.441613548 | down | chr19 | Map4k2 |
| 123 | mmu_circ_0008029 | circRNA | 0.0002022 | 2.906661619 | up | chr19 | Slc3a2 |
| 124 | mmu_circ_0008063 | circRNA | 0.001061015 | 0.389708839 | down | chr1 | Phlpp1 |
| 125 | mmu_circ_0008064 | circRNA | 3.73906E-05 | 0.389962461 | down | chr1 | Phlpp1 |
| 126 | mmu_circ_0009369 | circRNA | 0.002343379 | 0.243129436 | down | chr2 | Plcb1 |
| 127 | mmu_circ_0009373 | circRNA | 7.86393E-05 | 0.482703263 | down | chr2 | Plcb1 |
| 128 | mmu_circ_0010365 | circRNA | 9.48842E-06 | 0.378205757 | down | chr3 | Vav3 |
| 129 | mmu_circ_0013214 | circRNA | 0.015516266 | 3.084361224 | up | chr6 | Met |
| 130 | mmu_circ_0014859 | circRNA | 0.001034873 | 0.464785262 | down | chr8 | Vegfc |
| 131 | circRNA.9762 | circRNA | 0.000607668 | 2.385496546 | up | chr12 | Hif1a |
| 132 | circRNA.9760 | circRNA | 0.000403233 | 2.110220459 | up | chr12 | Hif1a |
| 133 | circRNA.9763 | circRNA | 0.000285925 | 2.28532873 | up | chr12 | Hif1a |
| 134 | circRNA.9761 | circRNA | 0.000849469 | 2.441186187 | up | chr12 | Hif1a |
| 135 | mmu_circ_0004178 | circRNA | 0.00159655 | 2.368707996 | up | chr12 | Hif1a |
| 136 | circRNA.13321 | circRNA | 0.011895344 | 3.36800166 | up | chr11 | Itgb3 |
| 137 | circRNA.25132 | circRNA | 0.001088707 | 0.479624152 | down | chr2 | Itgb6 |
| 138 | circRNA.25124 | circRNA | 0.012267938 | 0.454660539 | down | chr2 | Itgb6 |
| 139 | circRNA.25131 | circRNA | 0.000314864 | 0.453959803 | down | chr2 | Itgb6 |
| 140 | circRNA.25127 | circRNA | 0.001220071 | 0.343139529 | down | chr2 | Itgb6 |
| 141 | circRNA.25130 | circRNA | 0.001926767 | 0.410506973 | down | chr2 | Itgb6 |
| 142 | circRNA.25126 | circRNA | 0.005777434 | 0.465901288 | down | chr2 | Itgb6 |
| 143 | circRNA.24168 | circRNA | 1.68466E-05 | 0.33922876 | down | chr3 | Manba |
| 144 | circRNA.24161 | circRNA | 0.00075659 | 0.411847325 | down | chr3 | Manba |
| 145 | circRNA.24163 | circRNA | 0.000611272 | 0.457030896 | down | chr3 | Manba |
| 146 | circRNA.6587 | circRNA | 6.49913E-07 | 3.393059475 | up | chr14 | Flnb |
| 147 | circRNA.6518 | circRNA | 8.14357E-05 | 2.14721888 | up | chr14 | Flnb |
| 148 | circRNA.6520 | circRNA | 0.000381716 | 4.999703947 | up | chr14 | Flnb |
| 149 | circRNA.6534 | circRNA | 3.66866E-06 | 3.201618301 | up | chr14 | Flnb |
| 150 | circRNA.6537 | circRNA | 0.001441894 | 3.476521335 | up | chr14 | Flnb |
| 151 | circRNA.6526 | circRNA | 3.98486E-06 | 2.791131905 | up | chr14 | Flnb |
| 152 | circRNA.6585 | circRNA | 0.017731541 | 3.710624533 | up | chr14 | Flnb |
| 153 | mmu_circ_0005485 | circRNA | 0.002334498 | 6.489044773 | up | chr14 | Flnb |
| 154 | circRNA.6521 | circRNA | 2.90916E-06 | 4.366488739 | up | chr14 | Flnb |
| 155 | mmu_circ_0000501 | circRNA | 4.50439E-06 | 3.363725625 | up | chr14 | Flnb |
| 156 | circRNA.5573 | circRNA | 3.51666E-06 | 7.951490057 | up | chr15 | Osmr |
| 157 | circRNA.5562 | circRNA | 5.65242E-05 | 9.528418115 | up | chr15 | Osmr |
| 158 | circRNA.5563 | circRNA | 1.19437E-06 | 8.470842663 | up | chr15 | Osmr |
| 159 | circRNA.9280 | circRNA | 0.002920004 | 2.887430215 | up | chr13 | Pik3r1 |
| 160 | circRNA.26823 | circRNA | 0.000692793 | 0.372310309 | down | chr2 | Plcb1 |
| 161 | mmu_circ_0004399 | circRNA | 0.002667475 | 2.490403782 | up | chr13 | Pik3r1 |
| 162 | mmu_circ_0009374 | circRNA | 0.000131187 | 0.325759598 | down | chr2 | Plcb1 |
| 163 | mmu_circ_0005739 | circRNA | 8.76659E-06 | 9.071138659 | up | chr15 | Osmr |
| 164 | circRNA.5561 | circRNA | 0.000124535 | 2.653756514 | up | chr15 | Osmr |
| 165 | circRNA.13480 | circRNA | 4.7407E-05 | 3.043339446 | up | chr11 | Stat3 |
| 166 | circRNA.13477 | circRNA | 3.8878E-05 | 3.180671884 | up | chr11 | Stat3 |
| 167 | circRNA.27633 | circRNA | 0.000150866 | 2.308001675 | up | chr19 | Slc3a2 |
| 168 | circRNA.13484 | circRNA | 1.01623E-05 | 2.986995977 | up | chr11 | Stat3 |
| 169 | circRNA.13483 | circRNA | 4.10711E-06 | 3.651823589 | up | chr11 | Stat3 |
| 170 | circRNA.13479 | circRNA | 4.31095E-05 | 2.887582523 | up | chr11 | Stat3 |
| 171 | circRNA.27347 | circRNA | 0.000219792 | 3.570494678 | up | chr2 | Thbs1 |
| 172 | circRNA.13475 | circRNA | 9.30655E-05 | 2.938307122 | up | chr11 | Stat3 |
| 173 | circRNA.27350 | circRNA | 3.78596E-05 | 3.413379642 | up | chr2 | Thbs1 |

| | | | | | | | |
|---|---|---|---|---|---|---|---|
| 174 | circRNA.27354 | circRNA | 0.001627386 | 3.554465333 | up | chr2 | Thbs1 |
| 175 | mmu_circ_0010367 | circRNA | 0.000387088 | 0.354548548 | down | chr3 | Vav3 |
| 176 | circRNA.27353 | circRNA | 0.000169245 | 3.809606069 | up | chr2 | Thbs1 |
| 177 | mmu_circ_0010371 | circRNA | 0.001222222 | 0.359112464 | down | chr3 | Vav3 |
| 178 | circRNA.24392 | circRNA | 0.004966618 | 0.197732622 | down | chr3 | Vav3 |
| 179 | mmu_circ_0010369 | circRNA | 0.000152355 | 0.346638184 | down | chr3 | Vav3 |
| 180 | circRNA.24394 | circRNA | 7.23436E-05 | 0.314617656 | down | chr3 | Vav3 |
| 181 | mmu_circ_0010366 | circRNA | 8.09387E-06 | 0.378833199 | down | chr3 | Vav3 |
| 182 | mmu_circ_0010368 | circRNA | 2.03231E-05 | 0.311450725 | down | chr3 | Vav3 |
| 183 | circRNA.24393 | circRNA | 2.74781E-05 | 0.329755208 | down | chr3 | Vav3 |

**Table S3 All the differently expressed circRNAs involved in LPS-induced autophagy**

| Serial number | CircRNA_ID | Type | pvalues | Foldchange | Regulation | circ_chrom | Hostgene |
|---|---|---|---|---|---|---|---|
| 1 | circRNA.27393 | circRNA | 5.52283E-06 | 21.23872246 | up | chr2 | Pfkfb3 |
| 2 | circRNA.27392 | circRNA | 3.29012E-07 | 12.79919582 | up | chr2 | Pfkfb3 |
| 3 | circRNA.5564 | circRNA | 0.000628902 | 10.76847746 | up | chr15 | Osmr |
| 4 | circRNA.5562 | circRNA | 5.65242E-05 | 9.528418115 | up | chr15 | Osmr |
| 5 | circRNA.8088 | circRNA | 0.00027301 | 0.109760411 | down | chr13 | Rasgrf2 |
| 6 | mmu_circ_0005739 | circRNA | 8.76659E-06 | 9.071138659 | up | chr15 | Osmr |
| 7 | circRNA.5566 | circRNA | 3.23771E-06 | 8.707272797 | up | chr15 | Osmr |
| 8 | circRNA.5563 | circRNA | 1.19437E-06 | 8.470842663 | up | chr15 | Osmr |
| 9 | circRNA.27348 | circRNA | 0.004261081 | 8.175117047 | up | chr2 | Thbs1 |
| 10 | circRNA.5573 | circRNA | 3.51666E-06 | 7.951490057 | up | chr15 | Osmr |
| 11 | circRNA.27394 | circRNA | 1.22084E-07 | 7.136921458 | up | chr2 | Pfkfb3 |
| 12 | mmu_circ_0005485 | circRNA | 0.002334498 | 6.489044773 | up | chr14 | Flnb |
| 13 | circRNA.24429 | circRNA | 2.10381E-05 | 6.275860791 | up | chr3 | Csf1 |
| 14 | circRNA.19788 | circRNA | 1.37535E-05 | 0.164645979 | down | chr8 | Adcy7 |
| 15 | circRNA.1255 | circRNA | 1.30276E-05 | 5.594952363 | up | chr18 | Map3k8 |
| 16 | circRNA.24430 | circRNA | 3.21236E-05 | 5.502126901 | up | chr3 | Csf1 |
| 17 | circRNA.24392 | circRNA | 0.004966618 | 0.197732622 | down | chr3 | Vav3 |
| 18 | circRNA.6520 | circRNA | 0.000381716 | 4.999703947 | up | chr14 | Flnb |
| 19 | circRNA.24927 | circRNA | 0.003314941 | 0.200919678 | down | chr2 | Itga6 |
| 20 | circRNA.27121 | circRNA | 0.000278271 | 0.215429079 | down | chr2 | Itga8 |
| 21 | mmu_circ_0000837 | circRNA | 0.000126476 | 4.522719171 | up | chr18 | Map3k8 |
| 22 | circRNA.15204 | circRNA | 0.011705335 | 0.221742981 | down | chr10 | Ptprr |
| 23 | circRNA.13745 | circRNA | 0.003360385 | 4.472351657 | up | chr10 | Dram1 |
| 24 | circRNA.13324 | circRNA | 0.000125091 | 4.415431919 | up | chr11 | Itgb3 |
| 25 | circRNA.6521 | circRNA | 2.90916E-06 | 4.366488739 | up | chr14 | Flnb |
| 26 | circRNA.8466 | circRNA | 0.001804056 | 4.317688928 | up | chr13 | Gadd45g |
| 27 | circRNA.6522 | circRNA | 1.11223E-05 | 4.223497413 | up | chr14 | Flnb |
| 28 | circRNA.6581 | circRNA | 0.00043951 | 4.203408981 | up | chr14 | Flnb |
| 29 | mmu_circ_0007014 | circRNA | 2.372E-06 | 0.237983843 | down | chr17 | Rasgrp3 |
| 30 | circRNA.2845 | circRNA | 0.000142259 | 4.196161722 | up | chr17 | Daxx |
| 31 | mmu_circ_0002762 | circRNA | 0.002749617 | 0.239503933 | down | chr11 | Map2k6 |
| 32 | circRNA.2844 | circRNA | 3.84587E-05 | 4.132027996 | up | chr17 | Daxx |
| 33 | mmu_circ_0009369 | circRNA | 0.002343379 | 0.243129436 | down | chr2 | Plcb1 |
| 34 | circRNA.6519 | circRNA | 1.54914E-05 | 3.908359736 | up | chr14 | Flnb |
| 35 | circRNA.14328 | circRNA | 0.000111414 | 3.892722631 | up | chr10 | Ddit4 |
| 36 | circRNA.6586 | circRNA | 1.37981E-06 | 3.867151577 | up | chr14 | Flnb |
| 37 | circRNA.27353 | circRNA | 0.000169245 | 3.809606069 | up | chr2 | Thbs1 |
| 38 | circRNA.322 | circRNA | 0.002594915 | 3.712426602 | up | chr19 | Jak2 |
| 39 | circRNA.6585 | circRNA | 0.017731541 | 3.710624533 | up | chr14 | Flnb |
| 40 | circRNA.13483 | circRNA | 4.10711E-06 | 3.651823589 | up | chr11 | Stat3 |
| 41 | mmu_circ_0002861 | circRNA | 0.009202445 | 3.645048181 | up | chr11 | Egfr |
| 42 | circRNA.27347 | circRNA | 0.000219792 | 3.570494678 | up | chr2 | Thbs1 |
| 43 | circRNA.27354 | circRNA | 0.001627386 | 3.554465333 | up | chr2 | Thbs1 |
| 44 | mmu_circ_0004400 | circRNA | 0.000637095 | 3.535061943 | up | chr13 | Pik3r1 |
| 45 | circRNA.6537 | circRNA | 0.001441894 | 3.476521335 | up | chr14 | Flnb |
| 46 | circRNA.6523 | circRNA | 5.74056E-06 | 3.427690953 | up | chr14 | Flnb |
| 47 | circRNA.27350 | circRNA | 3.78596E-05 | 3.413379642 | up | chr2 | Thbs1 |
| 48 | circRNA.6587 | circRNA | 6.49913E-07 | 3.393059475 | up | chr14 | Flnb |
| 49 | circRNA.13321 | circRNA | 0.011895344 | 3.36800166 | up | chr11 | Itgb3 |
| 50 | circRNA.13481 | circRNA | 0.000130296 | 3.329259568 | up | chr11 | Stat3 |
| 51 | circRNA.17736 | circRNA | 2.64762E-06 | 3.277908041 | up | chr1 | Atp2b4 |
| 52 | circRNA.13476 | circRNA | 2.11202E-05 | 3.235220544 | up | chr11 | Stat3 |

| | | | | | | | | |
|---|---|---|---|---|---|---|---|---|
| 53 | circRNA.13121 | circRNA | 7.57718E-05 | 0.310619918 | down | chr11 | Map2k6 |
| 54 | mmu_circ_0010368 | circRNA | 2.03231E-05 | 0.311450725 | down | chr3 | Vav3 |
| 55 | circRNA.6534 | circRNA | 3.66866E-06 | 3.201618301 | up | chr14 | Flnb |
| 56 | mmu_circ_0004873 | circRNA | 0.006395832 | 0.313690283 | down | chr13 | Rasgrf2 |
| 57 | circRNA.13477 | circRNA | 3.8878E-05 | 3.180671884 | up | chr11 | Stat3 |
| 58 | circRNA.24394 | circRNA | 7.23436E-05 | 0.314617656 | down | chr3 | Vav3 |
| 59 | mmu_circ_0005484 | circRNA | 3.1078E-05 | 3.108091803 | up | chr14 | Flnb |
| 60 | circRNA.5560 | circRNA | 0.000100269 | 3.09544988 | up | chr15 | Osmr |
| 61 | mmu_circ_0013214 | circRNA | 0.015516266 | 3.084361224 | up | chr6 | Met |
| 62 | mmu_circ_0009374 | circRNA | 0.000131187 | 0.325759598 | down | chr2 | Plcb1 |
| 63 | circRNA.13480 | circRNA | 4.7407E-05 | 3.043339446 | up | chr11 | Stat3 |
| 64 | circRNA.24393 | circRNA | 2.74781E-05 | 0.329755208 | down | chr3 | Vav3 |
| 65 | mmu_circ_0001158 | circRNA | 1.32494E-05 | 0.33034386 | down | chr3 | Vav3 |
| 66 | circRNA.9488 | circRNA | 0.001008524 | 3.026753852 | up | chr12 | Fos |
| 67 | circRNA.24929 | circRNA | 0.000351418 | 0.331008476 | down | chr2 | Itga6 |
| 68 | circRNA.13484 | circRNA | 1.01623E-05 | 2.986995977 | up | chr11 | Stat3 |
| 69 | circRNA.13122 | circRNA | 0.000530375 | 0.335233513 | down | chr11 | Map2k6 |
| 70 | circRNA.24168 | circRNA | 1.68466E-05 | 0.33922876 | down | chr3 | Manba |
| 71 | circRNA.6533 | circRNA | 0.000661292 | 2.939091937 | up | chr14 | Flnb |
| 72 | circRNA.13475 | circRNA | 9.30655E-05 | 2.938307122 | up | chr11 | Stat3 |
| 73 | circRNA.12843 | circRNA | 2.43552E-05 | 2.928533307 | up | chr11 | Egfr |
| 74 | circRNA.25127 | circRNA | 0.001220071 | 0.343139529 | down | chr2 | Itgb6 |
| 75 | mmu_circ_0008029 | circRNA | 0.0002022 | 2.906661619 | up | chr19 | Slc3a2 |
| 76 | circRNA.13479 | circRNA | 4.31095E-05 | 2.887582523 | up | chr11 | Stat3 |
| 77 | circRNA.9280 | circRNA | 0.002920004 | 2.887430215 | up | chr13 | Pik3r1 |
| 78 | mmu_circ_0010369 | circRNA | 0.000152355 | 0.346638184 | down | chr3 | Vav3 |
| 79 | circRNA.25133 | circRNA | 0.001285103 | 0.347805182 | down | chr2 | Itgb6 |
| 80 | circRNA.13323 | circRNA | 0.000930612 | 2.848867785 | up | chr11 | Itgb3 |
| 81 | mmu_circ_0007907 | circRNA | 4.65809E-05 | 2.832508681 | up | chr19 | Rela |
| 82 | circRNA.13478 | circRNA | 4.64414E-05 | 2.83225849 | up | chr11 | Stat3 |
| 83 | circRNA.19972 | circRNA | 1.23071E-06 | 0.353443373 | down | chr8 | Vegfc |
| 84 | mmu_circ_0010367 | circRNA | 0.000387088 | 0.354548548 | down | chr3 | Vav3 |
| 85 | circRNA.6526 | circRNA | 3.98486E-06 | 2.791131905 | up | chr14 | Flnb |
| 86 | mmu_circ_0010371 | circRNA | 0.001222222 | 0.359112464 | down | chr3 | Vav3 |
| 87 | circRNA.12835 | circRNA | 5.222E-07 | 2.780695918 | up | chr11 | Egfr |
| 88 | circRNA.27819 | circRNA | 0.000536681 | 2.749574767 | up | chr19 | Rela |
| 89 | circRNA.6536 | circRNA | 0.006785795 | 2.705761008 | up | chr14 | Flnb |
| 90 | circRNA.13322 | circRNA | 0.04798225 | 2.691702596 | up | chr11 | Itgb3 |
| 91 | circRNA.8056 | circRNA | 0.003797045 | 0.371740907 | down | chr13 | Arsb |
| 92 | circRNA.18453 | circRNA | 3.73029E-05 | 2.674461162 | up | chrX | Timp1 |
| 93 | circRNA.27630 | circRNA | 0.000366252 | 2.670787787 | up | chr19 | Slc3a2 |
| 94 | circRNA.5561 | circRNA | 0.000124535 | 2.653756514 | up | chr15 | Osmr |
| 95 | mmu_circ_0010365 | circRNA | 9.48842E-06 | 0.378205757 | down | chr3 | Vav3 |
| 96 | mmu_circ_0010366 | circRNA | 8.09387E-06 | 0.378833199 | down | chr3 | Vav3 |
| 97 | circRNA.9281 | circRNA | 0.001089198 | 2.630031617 | up | chr13 | Pik3r1 |
| 98 | circRNA.21542 | circRNA | 0.001738668 | 0.380533108 | down | chr5 | Flt1 |
| 99 | circRNA.27968 | circRNA | 1.00316E-06 | 2.621704012 | up | chr19 | Nfkb2 |
| 100 | circRNA.9282 | circRNA | 0.002355879 | 2.614414444 | up | chr13 | Pik3r1 |
| 101 | circRNA.24600 | circRNA | 0.004541738 | 2.604438581 | up | chr2 | Creb3l1 |
| 102 | circRNA.6527 | circRNA | 3.71957E-06 | 2.599468016 | up | chr14 | Flnb |
| 103 | mmu_circ_0008063 | circRNA | 0.001061015 | 0.389708839 | down | chr1 | Phlpp1 |
| 104 | mmu_circ_0008064 | circRNA | 3.73906E-05 | 0.389962461 | down | chr1 | Phlpp1 |
| 105 | circRNA.12836 | circRNA | 9.26162E-06 | 2.562542184 | up | chr11 | Egfr |
| 106 | circRNA.2269 | circRNA | 0.010986647 | 0.39084646 | down | chr17 | Mtcl1 |
| 107 | circRNA.8463 | circRNA | 0.000244065 | 2.518636233 | up | chr13 | Syk |
| 108 | circRNA.26828 | circRNA | 0.000279382 | 0.399571084 | down | chr2 | Plcb1 |

| | | | | | | | |
|---|---|---|---|---|---|---|---|
| 109 | circRNA.7926 | circRNA | 0.000950812 | 0.399599599 | down | chr13 | Hexb |
| 110 | mmu_circ_0004874 | circRNA | 0.0041232 | 0.400908757 | down | chr13 | Rasgrf2 |
| 111 | mmu_circ_0004399 | circRNA | 0.002667475 | 2.490403782 | up | chr13 | Pik3r1 |
| 112 | circRNA.20257 | circRNA | 0.000271007 | 2.476654369 | up | chr7 | Dlg2 |
| 113 | circRNA.9761 | circRNA | 0.000849469 | 2.441186187 | up | chr12 | Hif1a |
| 114 | circRNA.25130 | circRNA | 0.001926767 | 0.410506973 | down | chr2 | Itgb6 |
| 115 | circRNA.24161 | circRNA | 0.00075659 | 0.411847325 | down | chr3 | Manba |
| 116 | circRNA.2387 | circRNA | 0.001071864 | 2.395763988 | up | chr17 | Efna5 |
| 117 | circRNA.9764 | circRNA | 0.001698145 | 2.387371881 | up | chr12 | Hif1a |
| 118 | circRNA.9762 | circRNA | 0.000607668 | 2.385496546 | up | chr12 | Hif1a |
| 119 | circRNA.18452 | circRNA | 9.64937E-06 | 2.37666495 | up | chrX | Timp1 |
| 120 | mmu_circ_0004178 | circRNA | 0.00159655 | 2.368707996 | up | chr12 | Hif1a |
| 121 | circRNA.24601 | circRNA | 0.000188848 | 2.349539595 | up | chr2 | Creb3l1 |
| 122 | circRNA.8464 | circRNA | 0.001378478 | 2.337143344 | up | chr13 | Syk |
| 123 | mmu_circ_0000079 | circRNA | 1.34915E-05 | 2.334737087 | up | chr1 | Atp2b4 |
| 124 | circRNA.25125 | circRNA | 0.002936208 | 0.429016876 | down | chr2 | Itgb6 |
| 125 | circRNA.27351 | circRNA | 0.000437367 | 2.328466029 | up | chr2 | Thbs1 |
| 126 | circRNA.11013 | circRNA | 0.007621395 | 0.432053407 | down | chr11 | Col1a1 |
| 127 | circRNA.27633 | circRNA | 0.000150866 | 2.308001675 | up | chr19 | Slc3a2 |
| 128 | circRNA.9763 | circRNA | 0.000285925 | 2.28532873 | up | chr12 | Hif1a |
| 129 | circRNA.13043 | circRNA | 6.92224E-05 | 2.270912816 | up | chr11 | Sphk1 |
| 130 | circRNA.12834 | circRNA | 0.000160538 | 2.263616592 | up | chr11 | Egfr |
| 131 | circRNA.15514 | circRNA | 0.001103255 | 0.442182055 | down | chr1 | Col6a3 |
| 132 | circRNA.12833 | circRNA | 0.00862962 | 2.250441241 | up | chr11 | Egfr |
| 133 | circRNA.6758 | circRNA | 0.000255368 | 2.249003256 | up | chr14 | Ppp2r2a |
| 134 | mmu_circ_0009458 | circRNA | 5.33878E-05 | 2.247745184 | up | chr2 | Chmp4b |
| 135 | circRNA.15516 | circRNA | 0.000743584 | 0.44778884 | down | chr1 | Col6a3 |
| 136 | circRNA.6757 | circRNA | 0.001063196 | 2.232888318 | up | chr14 | Ppp2r2a |
| 137 | circRNA.15367 | circRNA | 0.001296707 | 0.448901848 | down | chr10 | Kitl |
| 138 | circRNA.8376 | circRNA | 0.001608294 | 2.211191873 | up | chr13 | Ntrk2 |
| 139 | circRNA.11009 | circRNA | 0.001053621 | 0.452389882 | down | chr11 | Col1a1 |
| 140 | circRNA.3214 | circRNA | 0.003247997 | 0.452751748 | down | chr17 | Thbs2 |
| 141 | circRNA.3017 | circRNA | 0.002916199 | 0.453354432 | down | chr17 | Itpr3 |
| 142 | circRNA.27631 | circRNA | 0.007362063 | 2.204870165 | up | chr19 | Slc3a2 |
| 143 | circRNA.25131 | circRNA | 0.000314864 | 0.453959803 | down | chr2 | Itgb6 |
| 144 | circRNA.1946 | circRNA | 0.000620939 | 0.454093989 | down | chr17 | Map4k3 |
| 145 | circRNA.25124 | circRNA | 0.012267938 | 0.454660539 | down | chr2 | Itgb6 |
| 146 | circRNA.24163 | circRNA | 0.000611272 | 0.457030896 | down | chr3 | Manba |
| 147 | circRNA.24162 | circRNA | 0.000205665 | 0.457668418 | down | chr3 | Manba |
| 148 | circRNA.6843 | circRNA | 0.000160591 | 0.457965038 | down | chr14 | Gata4 |
| 149 | circRNA.6583 | circRNA | 8.21105E-05 | 2.178283139 | up | chr14 | Flnb |
| 150 | circRNA.24166 | circRNA | 5.17069E-05 | 0.462661468 | down | chr3 | Manba |
| 151 | circRNA.6756 | circRNA | 0.001159814 | 2.160484474 | up | chr14 | Ppp2r2a |
| 152 | mmu_circ_0014859 | circRNA | 0.001034873 | 0.464785262 | down | chr8 | Vegfc |
| 153 | circRNA.12839 | circRNA | 0.000264697 | 2.150247089 | up | chr11 | Egfr |
| 154 | circRNA.6518 | circRNA | 8.14357E-05 | 2.14721888 | up | chr14 | Flnb |
| 155 | circRNA.25126 | circRNA | 0.005777434 | 0.465901288 | down | chr2 | Itgb6 |
| 156 | circRNA.8170 | circRNA | 0.00026631 | 0.468454063 | down | chr13 | Mef2c |
| 157 | circRNA.12838 | circRNA | 0.00150193 | 2.134456089 | up | chr11 | Egfr |
| 158 | circRNA.25129 | circRNA | 0.00203312 | 0.468964405 | down | chr2 | Itgb6 |
| 159 | circRNA.24164 | circRNA | 0.000505164 | 0.469138253 | down | chr3 | Manba |
| 160 | mmu_circ_0004891 | circRNA | 0.001061728 | 0.469845821 | down | chr13 | Arsb |
| 161 | mmu_circ_0002648 | circRNA | 4.09575E-05 | 0.472349674 | down | chr11 | Dusp3 |
| 162 | mmu_circ_0004829 | circRNA | 0.000446071 | 0.472547038 | down | chr13 | Mef2c |
| 163 | circRNA.2818 | circRNA | 0.004813006 | 0.472764223 | down | chr17 | Tnxb |
| 164 | circRNA.13997 | circRNA | 6.2051E-06 | 0.473145352 | down | chr10 | Col6a1 |

| | | | | | | | |
|---|---|---|---|---|---|---|---|
| 165 | circRNA.9760 | circRNA | 0.000403233 | 2.110220459 | up | chr12 | Hif1a |
| 166 | circRNA.15513 | circRNA | 0.000153429 | 0.47427428 | down | chr1 | Col6a3 |
| 167 | circRNA.27349 | circRNA | 0.004210571 | 2.108062453 | up | chr2 | Thbs1 |
| 168 | circRNA.21541 | circRNA | 6.83307E-05 | 0.47798115 | down | chr5 | Flt1 |
| 169 | circRNA.25132 | circRNA | 0.001088707 | 0.479624152 | down | chr2 | Itgb6 |
| 170 | circRNA.11010 | circRNA | 0.001131877 | 0.480234958 | down | chr11 | Col1a1 |
| 171 | mmu_circ_0004830 | circRNA | 0.001073782 | 0.480439408 | down | chr13 | Mef2c |
| 172 | mmu_circ_0009373 | circRNA | 7.86393E-05 | 0.482703263 | down | chr2 | Plcb1 |
| 173 | mmu_circ_0004176 | circRNA | 0.000424237 | 2.059978721 | up | chr12 | Hif1a |
| 174 | circRNA.9809 | circRNA | 6.18738E-05 | 2.057132018 | up | chr12 | Sav1 |
| 175 | circRNA.13749 | circRNA | 7.10782E-05 | 0.486231614 | down | chr10 | Igf1 |
| 176 | circRNA.9796 | circRNA | 0.001169277 | 0.490266149 | down | chr12 | Frmd6 |
| 177 | circRNA.1953 | circRNA | 0.002407971 | 0.494190526 | down | chr17 | Map4k3 |
| 178 | mmu_circ_0005852 | circRNA | 6.48978E-05 | 2.009153381 | up | chr15 | Atf4 |
| 179 | circRNA.12832 | circRNA | 0.001480865 | 2.005143483 | up | chr11 | Egfr |

# Original Blots

## Original Blots for Fig.5

### Fig.5A

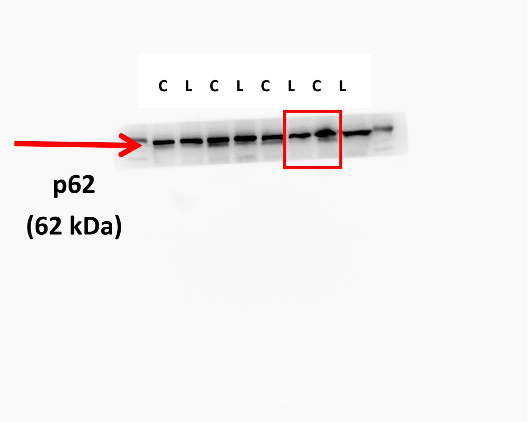


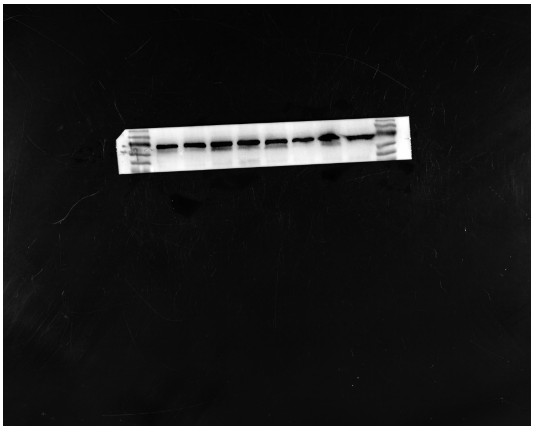

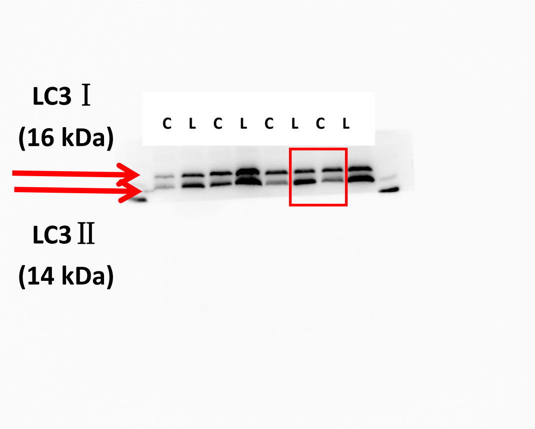


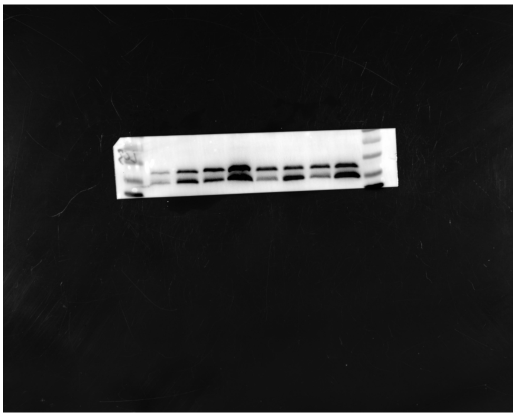

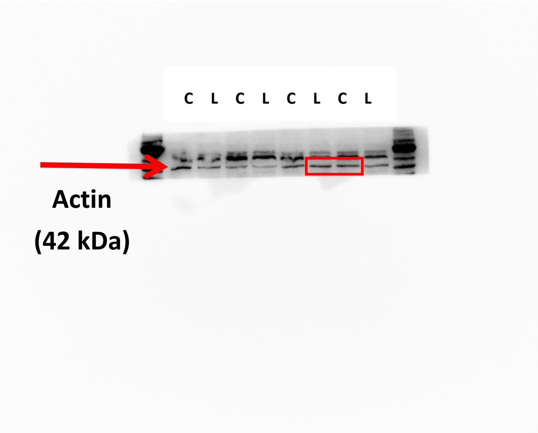


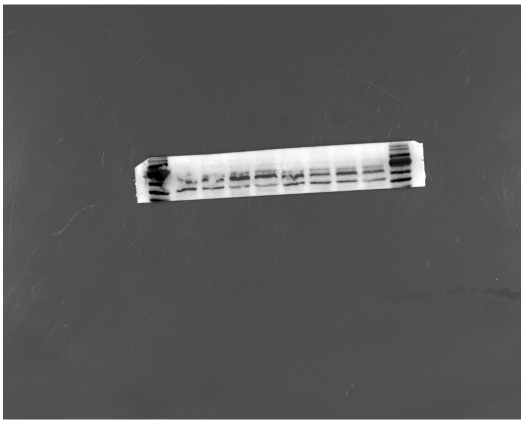

C = Ctrl L = LPS
